# Source space connectomics of neurodegeneration: One-metric approach does not fit all

**DOI:** 10.1016/j.nbd.2023.106047

**Published:** 2023-02-23

**Authors:** Pavel Prado, Sebastian Moguilner, Jhony A. Mejía, Agustín Sainz-Ballesteros, Mónica Otero, Agustina Birba, Hernando Santamaria-Garcia, Agustina Legaz, Sol Fittipaldi, Josephine Cruzat, Enzo Tagliazucchi, Mario Parra, Rubén Herzog, Agustín Ibáñez

**Affiliations:** a Latin American Brain Health Institute (BrainLat), Universidad Adolfo Ibáñez, Santiago, Chile; b Escuela de Fonoaudiología, Facultad de Odontología y Ciencias de la Rehabilitación, Universidad San Sebastián, Santiago, Chile; c Cognitive Neuroscience Center (CNC), Universidad de San Andrés & CONICET, Buenos Aires, Argentina; d Departamento de Ingeniería Biomédica, Universidad de Los Andes, Bogotá, Colombia; e Facultad de Ingeniería, Arquitectura y Diseño, Universidad San Sebastián, Santiago, Chile; f Centro BASAL Ciencia & Vida, Universidad San Sebastián, Santiago, Chile; g PhD Neuroscience Program, Physiology and Psychiatry Departments, Pontificia Universidad Javeriana, Bogotá, Colombia; h Memory and Cognition Center Intellectus, Hospital Universitario San Ignacio, Bogotá, Colombia; i Global Brain Health Institute, University of California San Francisco, San Francisco, California; j Global Brain Health Institute, Trinity College Dublin, Dublin, Ireland; k National Scientific and Technical Research Council, Buenos Aires, Argentina; l Departamento de Física, Universidad de Buenos Aires and Instituto de Física de Buenos Aires (IFIBA -CONICET), Buenos Aires, Argentina; m School of Psychological Sciences and Health, University of Strathclyde, Glasgow, United Kingdom; n Fundación para el Estudio de la Conciencia Humana (EcoH), Chile.; o Trinity College Dublin (TCD), Dublin, Ireland

**Keywords:** Composite connectivity metric, Connectomics, Dementia biomarker, EEG source-space, Multi-feature machine learning classification

## Abstract

Brain functional connectivity in dementia has been assessed with dissimilar EEG connectivity metrics and estimation procedures, thereby increasing results’ heterogeneity. In this scenario, joint analyses integrating information from different metrics may allow for a more comprehensive characterization of brain functional interactions in different dementia subtypes. To test this hypothesis, resting-state electroencephalogram (rsEEG) was recorded in individuals with Alzheimer’s Disease (AD), behavioral variant frontotemporal dementia (bvFTD), and healthy controls (HCs). Whole-brain functional connectivity was estimated in the EEG source space using 101 different types of functional connectivity, capturing linear and nonlinear interactions in both time and frequency-domains. Multivariate machine learning and progressive feature elimination was run to discriminate AD from HCs, and bvFTD from HCs, based on joint analyses of i) EEG frequency bands, ii) complementary frequency-domain metrics (e.g., instantaneous, lagged, and total connectivity), and iii) time-domain metrics with different linearity assumption (e.g., Pearson correlation coefficient and mutual information). <10% of all possible connections were responsible for the differences between patients and controls, and atypical connectivity was never captured by >1/4 of all possible connectivity measures. Joint analyses revealed patterns of hypoconnectivity (patients<HCs) involving convergent temporo-parieto-occipital regions in AD, and fronto-temporo-parietal areas in bvFTD. Hyperconnectivity (patients>HCs) in both groups was mainly identified in frontotemporal regions. These atypicalities were differently captured by frequency- and time-domain connectivity metrics, in a bandwidth-specific fashion. The multi-metric representation of source space whole-brain functional connectivity evidenced the inadequacy of single-metric approaches, and resulted in a valid alternative for the selection problem in EEG connectivity. These joint analyses reveal patterns of brain functional interdependence that are overlooked with single metrics approaches, contributing to a more reliable and interpretable description of atypical functional connectivity in neurodegeneration.

## 1. Introduction

Alzheimer’s disease (AD) and behavioral variant frontotemporal dementia (bvFTD) are neurodegenerative conditions urgently calling for affordable, scalable, cross-cultural, and physio-pathological plausible biomarkers accompanying diagnosis and characterization (Dubois et al., 2021; Jagust, 2021; Parra et al., 2020; Migeot et al., 2022). These diseases involve neurodegeneration derived from protein aggregates (Ferrari and Sorbi, 2021; Peet et al., 2021) that dysregulate the synaptic transmission and disturb the oscillatory brain dynamic (Martorell et al., 2019; Palop and Mucke, 2010). Since brain rhythms reflect the communication and information transfer in distributed cortical networks (Buzsáki and Draguhn, 2004), brain functional connectivity constitutes a potential target for dementia characterization (Yu et al., 2021; Zhao et al., 2022).

Functional connectivity in neurodegeneration has been mostly tackled through magnetic resonance imaging (MRI). Nevertheless, high-density electroencephalography (hdEEG) has recently emerged as an alternative, scalable, and cost-effective benchmark (Babiloni et al., 2021; Law et al., 2020; Livinț Popa et al., 2021). The hdEEG offers a direct measure of neural activity with high temporal resolution, and therefore allows for the accurate assessment of whole-brain oscillatory dynamics and network disintegration (Goriely et al., 2020; Rossini et al., 2020). Consequently, the hdEGG has been posited as a physiological dementia biomarker (Babiloni et al., 2020, 2021) that may provide additional and supplementary relevant information to that gathered by A (amyloid), T (tau) and N (neurodegeneration) biomarkers (Jack Jr et al., 2018).

Notwithstanding the potential use of EEG for dementia characterization, different approaches to brain functional connectivity yield divergent results depending on whether they consider whole-brain or local analysis. Furthermore, the topographic information of brain functional interactions depends on whether connectivity analyses are conducted on scalp-recorded signals or source-space transformed EEG (Babiloni et al., 2016, 2018; San-Martin et al., 2021). Most importantly, disparate results are accounted for by the wide variety of connectivity metrics available (Briels et al., 2020; Gaubert et al., 2019; Musaeus et al., 2019). Since different metrics reflect different types of functional interactions (Mohanty et al., 2020; Prado et al., 2022), generalizations based on a single measure may not be feasible. Instead, a combination of metrics may be critical to refining the hypothesis space of functional interactions in neurodegeneration.

Since a complete overview of functional connectivity may result from the integration of information captured by different connectivity metrics, the goal of this study is testing the efficacy of using different composite metric of functional connectivity for dementia characterization. We hereby propose a comprehensive multi-metric analysis of source space EEG connectivity of AD and bvFTD. We hypothesize that joint analyses integrating complementary information gathered by a large set of connectivity metrics provide an all-encompassing approach to whole-brain functional connectivity and offer a comprehensive set of linear and nonlinear brain functional interactions (in both frequency and time domains) to accurately classify dementia (e.g., classify AD from HCs, and bvFTD from HCs). To test this hypothesis, we acquired rsEEG in AD, bvFTD, and elderly healthy control (HCs) individuals. One hundred and one types of functional connectivity were computed from source-localized resting estate EEG (rsEEG), which considered linear and nonlinear interactions in both time- and frequency-domains. Machine learning classifiers combined with progressive feature elimination procedures were run to discriminate each dementia subtype from healthy controls. We predicted that robust classifications of either AD or bvFTD relative to HCs result from integrating different i) frequency bands, ii) complementary frequency-domain metrics (e.g., instantaneous, lagged, and total coherence), and iii) time-domain metrics with different linearity assumption. These predictions may support a comprehensive whole-brain functional connectivity approach that integrates source space EEG interactions in different domains to accurately represent the disintegration of functional networks in neurodegeneration.

## 2. Methods

### 2.1. Participants

Ninety-four individuals were enrolled in this study, in clinical sites from the Multi-Partner Consortium to Expand Dementia Research in Latin America (ReDLat) (Ibanez et al., 2021a, 2021b), a regional effort aimed at the harmonization of participant enrollment and neurocognitive assessment in multicentric studies (Birba et al., 2022; Legaz et al., 2022; Salamone et al., 2021). Participants belonged to one of three groups: patients with Alzheimer’s Disease (AD, *n* = 33), patients with behavioral variant frontotemporal dementia (bvFTD, *n* = 19), and elderly healthy control individuals (HCs, *n* = 42). Current international NINCDS-ADRDA criteria for AD (McKhann et al., 2011) as well as the revised criteria for probable bvFTD (Rascovsky et al., 2011) were used for diagnosis, which was also supported by extensive neurological, neuropsychiatric, and neuropsychological examination (Table 1). All patients were in the early/mild stages of the disease and had no proven track of substance abuse, primary language deficits, nor neurological or psychiatric disorders.

The age, sex, and years of formal education of each group of patients were comparable with those of HCs (Table 1). The sample size of the study and the number of individuals who belonged to each group was larger than the minimum sample necessary to conduct F-tests (one-way ANOVA) sensitive to large effect sizes with a statistical power of 0.8. Likewise, the sample was large enough to conduct a two-tailed *t*-test, able to sense large effect sizes with a statistical power of 0.8. This study was approved by the institutional reviewer board of each recruitment site. Before enrolling, a signed informed consent was provided by all participants following the Declaration of Helsinki.

### 2.2. Acquisition and processing

**Fig. 1.**
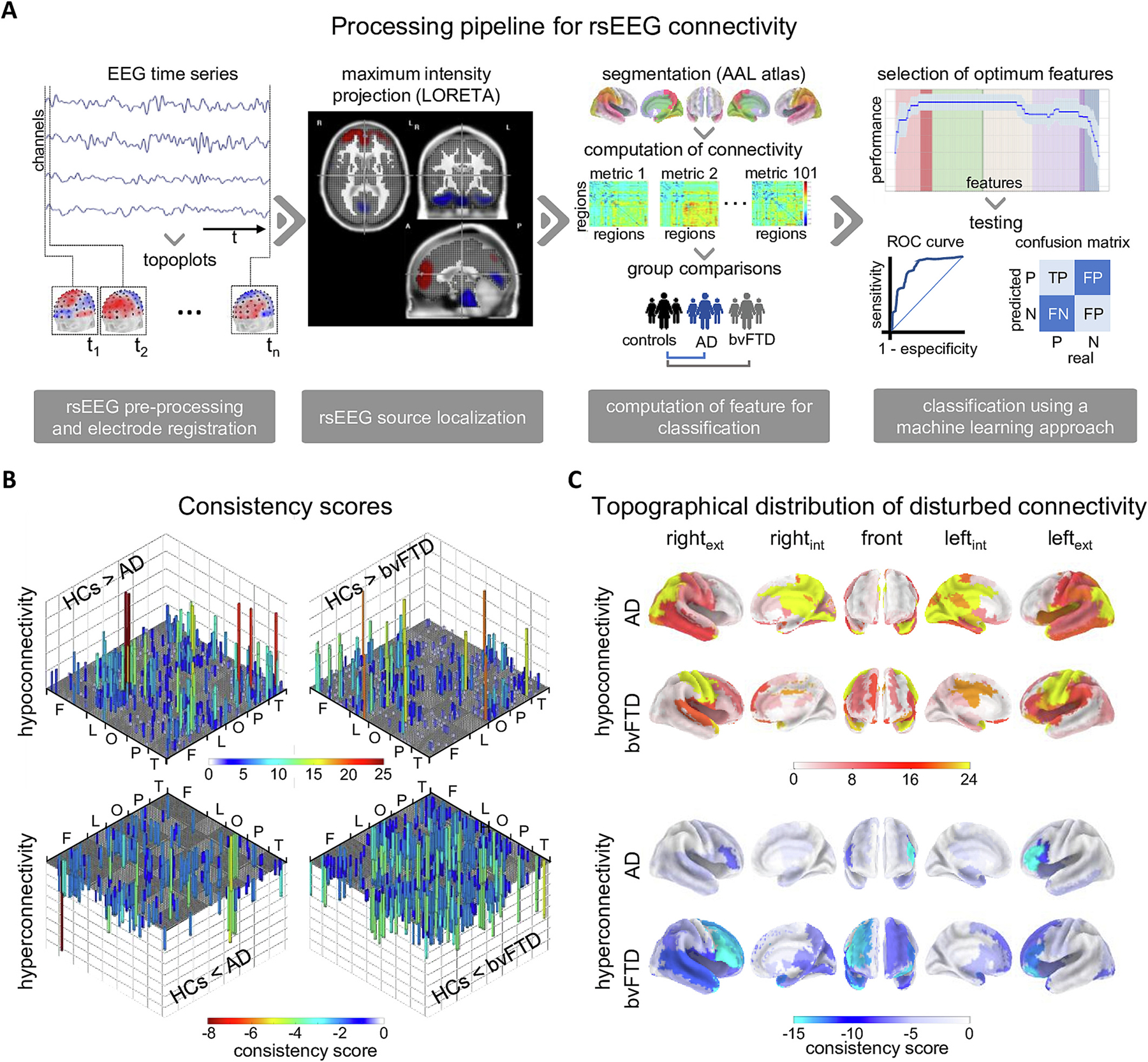
An integrative approach to whole-brain functional connectivity in dementia A) Workflow for the classification of dementia subtypes. The pipeline includes signal processing steps that are critical for the harmonization of rsEEG in multicentric studies. The Low-Resolution Electromagnetic Tomography (LORETA) was used for rsEEG source localization. A total of 101 representations of whole-brain functional connectivity were obtained and integrated for the classification of Alzheimer’s disease (AD), and behavioral variant frontotemporal dementia (bvFTD) relative to elderly healthy indoviduals (HCs) B) Consistency score of pairwise connections with statistically significant differences between each dementia subtype (AD, and bvFTD) and HCs. The consistency score (the number of connectivity metrics to which the pairwise connection differed between groups) is represented by the size and color of the vertical bars. Regions were sorted following the organization of the automatic anatomical labeling (AAL) atlas. Different brain lobes are indicated (F: frontal, L: limbic, O: occipital, P: parietal, T: temporal). Hypoconnectivity and hyperconnectivity in dementia are presented in top and lower panels, respectively. C) Topographical distribution of regions with atypical functional connectivity in AD, and bvFTD. Regions with decreased and increased connectivity in comparison to HCs are presented in the top and lower panels, respectively.

Participants sat in a comfortable chair inside a dimly lit, sound-attenuated and electromagnetically shielded EEG chamber. Following previous EEG studies in dementia (Dottori et al., 2017; Birba et al., 2022), individuals were instructed to remain still and awake. Ongoing (resting-state), eyes-closed EEG was recorded for five minutes using a Biosemi Active-two acquisition system with 128 scalp electrodes (a high-density electrode layout), in a radial configuration. The reference electrodes were set to linked mastoids. Electrodes were also placed in periocular locations to record blinks and eye movements. Analog filters were set at 0.03 and 100 Hz. During the analog/digital conversion, signals were sampled at 1024 Hz, with 24 bits of resolution. The EEG was processed offline using procedures implemented in a custom, automatic pipeline, which is described in Fig. 1A.

Recordings were re-referenced to the average reference, and band-pass filtered between 0.5 and 40 Hz using a zero-phase shift Butterworth filter of order = 8. Data were down-sampled to 512 Hz, and Independent Component Analysis (ICA) was used to correct EEG artifacts induced by blinking and eye movements (Delorme and Makeig, 2004). Malfunctioning channels were identified and replaced using weighted spherical interpolation. Following guidelines for multicentric studies (Prado et al., 2022), patient-control normalizations were conducted to decrease inter-subject variability (Bigdely-Shamlo et al., 2020).

### 2.3. Source localization of rsEEG

The source analysis of the rsEEG was conducted using the standardized Low-Resolution Electromagnetic Tomography method (sLORETA, Pascual-Marqui, 2002; for a review, see Grech et al., 2008). sLORETA allows estimating the standardized current density at each of the predefined virtual sensors located in the cortical grey matter and the hippocampus of a reference brain (MNI 305, Brain Imaging Centre, Montreal Neurologic Institute) based on the linear, weighted sum of a particular scalp voltage distribution or the EEG cross-spectrum at the sensor level. sLORETA is based on an appropriately standardized version of the minimum norm current density estimation which overcomes problems intrinsic to the estimation of deep sources of EEG (Asadzadeh et al., 2020; Bradley et al., 2016).

The Biosemi 128 electrode layout was registered onto the scalp MNI152 coordinates. Landmarks for registering the Biosemi electrode locations were Nasion, Inion, and both left and right preauricular points. The location of landmarks and recording electrodes were expressed in millimeters, using the Cartesian coordinate system. A signal-to-noise ratio of 1 was the choice for the regularization method used to compute the sLORETA transformation matrix (forward operator for the inverse solution problem).

The standardized current density maps were obtained using a head model of three concentric spheres, in a predefined source space of 6242 voxels (voxel size of 5mm^3^) of the MNI average brain. A brain segmentation of 82 anatomic compartments (cortical areas) was implemented using the automated anatomical labeling (AAL90) atlas (Rolls et al., 2015). The list of the AAL regions and the abbreviations used for identification are provided in Table S1. Current densities were estimated for each of the 153,600 voltage distributions comprising the five-minutes of rsEEG (sampled at 512 Hz) and they were averaged among voxels belonging to the same AAL region, such that a single (mean) time series was obtained for each cortical region (Herzog et al., 2022; Cruzat et al., 2023).

### 2.4. Connectivity metrics

Whole-brain functional connectivity was analyzed using 101 types of functional interactions. The selection covered both linear and nonlinear functional connectivity metrics (Table 2). Five metrics provided information on time-domain interdependencies, while four metrics informed about frequency-domain interdependencies. Each metric in the frequency-domain considered instantaneous, lagged, and total connectivity, which will be referred to as a set of complementary connectivity metric throughout this document. They were computed in eight EEG frequency bands: delta (*δ*: 1.5–4 Hz), theta (*θ*: 4–8 Hz), alpha1 (*α*1: 8–10 Hz), alpha2 (*α*2: 10–13 Hz), beta1 (*β*1: 13–18 Hz), beta2 (*β*2: 18–21 Hz), beta3 (*β*3: 21–30 Hz), and gamma (*γ*: 30–40 Hz). Therefore, 96 types of functional interactions were analyzed in the frequency-domain (4 metrics × 3 complementary connectivity metrics x 8 EEG frequency bands). Adding the five time-domain connectivity metrics, we obtained the total of 101 types of functional interactions.

#### 2.4.1. Frequency-domain metrics

Functional connectivity in the frequency-domain was computed using Loreta Key (Pascual-Marqui, 1999, 2002; Pascual-Marqui et al., 1994), which allows for the estimation of linear and nonlinear statistical dependencies of the electrical activity of neurons between several brain locations (Pascual-Marqui, 2007b). For simplicity, equations are presented for the case of two multivariate time series. Metrics included in the study are presented below.

##### 2.4.1.1. Linear connectivity (LC).

The LC measures the linear dependency between multivariate time series at a given frequency (ω) (Pascual-Marqui, 2007a). Computation is based on their respective complex valued covariance matrix (S). Since S is obtained from the discrete Fourier transform, LC conveys information about both amplitude and phase of the oscillatory activity. For a pair of multivariate time series x, and y, the LC (F) at the frequency ω can be expressed as.

(1) $$F_{x,y}(\omega )=ln\frac{\left|\left(\begin{array}{cc} S_{yy(\omega )} & 0\\ 0^{t} & S_{yy(\omega )} \end{array}\right)\right|}{\left|\left(\begin{array}{ll} S_{yy(\omega )} & S_{yx(\omega )}\\ S_{xy(\omega )} & S_{xx(\omega )} \end{array}\right)\right|}$$

where |M| represents the determinant of the matrix M, and t denotes time.

The measure $F_{x,y}(\omega )$ can be expressed as the linear combination of the instantaneous LC (instLC), and the lagged LC (laggedLC) between time series, such that the total LC (totalLC) can be expressed as.

(2) $$F_{x,y}(\omega )=F_{x⇄y}(\omega )+F_{x•y}$$

The instLC (Fxy(ω)) can be expressed as.

(3) $$F_{x•y}(\omega )=ln\frac{\left|Re\left(\begin{array}{cc} S_{yy(\omega )} & 0\\ 0^{t} & S_{xx(\omega )} \end{array}\right)\right|}{\left|Re\left(\begin{array}{cc} S_{yy(\omega )} & S_{yx(\omega )}\\ S_{xy(\omega )} & S_{xx(\omega )} \end{array}\right)\right|}$$

where Re(M) denotes the real part of M. All three measures (instLC, laggedLC, and totalLC) are non-negative, such that zero values indicate no dependence between the time series.

##### 2.4.1.2. Coherence (Coh).

The Coh refers to a squared coherence type measure and represent an estimate of the consistency of relative amplitude and phase between signals, taking values between 0 and 1 (0 indicates no coherence and 1 indicates maximum coherence between the signals) (Pascual-Marqui, 2007a). The general Coh can be expressed as.


(4) $$\rho_{x,y}^{2}(\omega )=1-exp\left(-F_{x,y}(\omega )\right)$$

In the case of two multivariate time series (x,y) the instantaneous Coh (instCoh) is related to the real part of the complex valued coherency, and can be defined as.


(5) $$\rho_{x•y}^{2}(\omega )=1-exp\left[-F_{x,y}(\omega )\right]=1-\frac{\left|Re\left(\begin{array}{ll} S_{yy(\omega )} & S_{yx(\omega )}\\ S_{xy(\omega )} & S_{xx(\omega )} \end{array}\right)\right|}{\left|Re\left(\begin{array}{cc} S_{yy(\omega )} & 0\\ 0^{t} & S_{xx(\omega )} \end{array}\right)\right|}$$

The lagged Coh (laggedCoh) is given by the expression.


(6) $$\rho_{x⇄y}^{2}(\omega )\;=1-exp\left[-F_{x⇆y}(\omega )\right]\;\;=-\frac{\left\{\left|\left(\begin{array}{cc} S_{yy(\omega )} & S_{yx(\omega )}\\ S_{xy(\omega )} & S_{xx(\omega )} \end{array}\right)\right|/\left|\left(\begin{array}{cc} S_{yy(\omega )} & 0\\ 0^{t} & S_{xx(\omega )} \end{array}\right)\right|\right\}}{\left\{\left|Re\left(\left(\begin{array}{cc} S_{yy(\omega )} & S_{xy(\omega )}\\ S_{xy(\omega )} & S_{xx(\omega )} \end{array}\right)\right)\right|/\left|Re\left(\begin{array}{cc} S_{yyy} & 0\\ 0^{t} & S_{xx\omega } \end{array}\right)\right|\right\}}$$

The laggedCoh $\left(\rho^{2}(\omega )\right)$ represents a dependence measure that is not affected by the covariance structure of each time series. Therefore, laggedCoh can be considered a reliable measure of functional connectivity since it is not sensitive to the confounding effect of volume conduction and low spatial resolution inherent to the solution of the inverse problem in EEG (Pascual-Marqui, 2007a). Noteworthy, totalCoh cannot be expressed as the sum of instCoh, and laggedCoh (Pascual-Marqui, 2007b).

##### 2.4.1.3. Non-linear connectivity (nLC).

The nLC measures the nonlinear dependency between multivariate time series (x,y) at the frequency ω (Pascual-Marqui, 2007a). Since S is obtained from the normalized discrete Fourier transform in which amplitude information is factored out, nLC only comprises information about the phase of the oscillatory activity. For the case of two time series, the total nLC (totalnLC) satisfies the relation.

(7) $$G_{x,y}(\omega )=G_{x•y}(\omega )+G_{x⇄y}(\omega )$$

where Gxy(ω), and Gx→y(ω) represent the instantaneous nLC (instnLC) and lagged nLC (laggednLC), respectively. The totalnLC (Gx,(ω))) and instnLC (Gx→y(ω)) linear connectivity can be expressed as.

(8) $$G_{x,y}(\omega )=ln\frac{\left|\left(\begin{array}{cc} S_{\overset{˘}{y}\overset{˘}{y}(\omega )} & 0\\ 0^{t} & S_{\overset{˘}{x}\overset{˘}{x}(\omega )} \end{array}\right)\right|}{\left|\left(\begin{array}{cc} S_{\overset{˘}{y}\overset{˘}{y}(\omega )} & S_{\overset{˘}{y}\overset{˘}{x}(\omega )}\\ S_{\overset{˘}{x}\overset{˘}{y}(\omega )} & S_{\overset{˘}{x}\overset{˘}{x}(\omega )} \end{array}\right)\right|}$$

and

(9) $$G_{x•y}(\omega )=ln\frac{\left|Re\left(\begin{array}{cc} S_{\overset{˘}{y}\overset{˘}{y}(\omega )} & 0\\ 0^{t} & S_{\overset{˘}{x}\overset{˘}{x}(\omega )} \end{array}\right)\right|}{\left|Re\left(\begin{array}{cc} S_{\overset{˘}{y}\overset{˘}{y}(\omega )} & S_{\overset{˘}{y}\overset{˘}{x}(\omega )}\\ S_{\overset{˘}{x}\overset{˘}{y}(\omega )} & S_{\overset{˘}{x}\overset{˘}{x}(\omega )} \end{array}\right)\right|}$$

where Re(M) denotes the real part of M, and t denotes time. All three measures (laggednLC, instnLC, or totalnLC) are non-negative, such that zero values indicate no dependence between the time series.

##### 2.4.1.4. Phase synchronization (PS).

The PS measures the stability of the phase difference between a group of multivariate time series at a particular frequency ω, regardless the amplitude information (Pascual-Marqui, 2007a). Phase synchronization is equivalent to phase-locking value, phase-locking index, and phase coherence, among others. For the computation of PS, the Fourier transforms are normalized before the coherence calculation to rule out any amplitude information. Since it is represented as the absolute value of the complex valued coherency between the normalized Fourier transforms, it bounds between zero (no synchronization) to one (perfect synchronization). In the case of two time series (x,y), the total PS (totalPS), denoted by $\varphi_{x,y}^{2}$, is given by the expression.


(10) $$\varphi_{x,y}^{2}(\omega )=1-exp\left(-G_{x,y}(\omega )\right)=1-\frac{\left|\left(\begin{array}{cc} S_{\overset{˘}{y}\overset{˘}{y}(\omega )} & S_{\overset{˘}{y}\overset{˘}{x}(\omega )}\\ S_{\overset{˘}{x}\overset{˘}{y}(\omega )} & S_{\overset{˘}{x}\overset{˘}{x}(\omega )} \end{array}\right)\right|}{\left|\left(\begin{array}{cc} S_{\overset{˘}{y}\overset{˘}{y}(\omega )} & 0\\ 0^{t} & S_{\overset{˘}{x}\overset{˘}{x}(\omega )} \end{array}\right)\right|}$$

The instantaneous PS (instPS) and lagged PS (laggedPS) can be expressed as.

(11) $$\varphi_{x•y}^{2}(\omega )=1-exp\left(-G_{x•y}(\omega )\right)=1-\frac{\left|Re\left(\begin{array}{cc} S_{\overset{˘}{y}\overset{˘}{y}(\omega )} & S_{\overset{˘}{y}\overset{˘}{x}(\omega )}\\ S_{\overset{˘}{x}\overset{˘}{y}(\omega )} & S_{\overset{˘}{x}\overset{˘}{x}(\omega )} \end{array}\right)\right|}{\left|Re\left(\begin{array}{cc} S_{\overset{˘}{y}\overset{˘}{y}(\omega )} & 0\\ 0^{t} & S_{\overset{˘}{x}\overset{˘}{x}(\omega )} \end{array}\right)\right|}$$

and

(12) $$\varphi_{x⇄y}^{2}(\omega )=1-exp\left[-G_{x⇆y}(\omega )\right]\;\;=-\frac{\left\{\left|\left(\begin{array}{cc} S_{\overset{˘}{y}\overset{˘}{y}(\omega )} & S_{\overset{˘}{y}\overset{˘}{x}(\omega )}\\ S_{\overset{˘}{x}\overset{˘}{y}(\omega )} & S_{\overset{˘}{x}\overset{˘}{x}(\omega )} \end{array}\right)\right|/\left|\left(\begin{array}{cc} S_{\overset{˘}{y}\overset{˘}{y}(\omega )} & 0\\ 0^{t} & S_{\overset{˘}{x}\overset{˘}{x}(\omega )} \end{array}\right)\right|\right\}}{\left\{\left|Re\left(\begin{array}{cc} S_{\overset{˘}{\overset{˘}{y}y}(\omega )} & S_{\overset{˘}{y}\overset{˘}{x}(\omega )}\\ S_{\overset{˘}{x}\overset{˘}{y}(\omega )} & S_{\overset{˘}{x}\overset{˘}{x}(\omega )} \end{array}\right)\right|/\left|Re\left(\begin{array}{cc} S_{\overset{˘}{y}\overset{˘}{y}\omega } & 0\\ 0^{t} & S_{\overset{˘}{x}\overset{˘}{x}(\omega )} \end{array}\right)\right|\right\}}$$

respectively, where totalPS $\left(\varphi_{x,y}^{2}(\omega )\right)$ cannot be expressed as the sum of instPS $\left(\varphi_{x•y}^{2}(\omega )\right)$, and laggedPS $\left({\varphi_{x⥄y}}^{2}\left(\omega \right)\right)$ (Pascual-Marqui, 2007b). The laggedPS provides a measure of functional connectivity that is not affected by the volume conduction and low spatial resolution inherent to the solution of the inverse problem in EEG (Pascual-Marqui, 2007a).

#### 2.4.2. Time-domain metrics

##### 2.4.2.1. Pearson’s correlation coefficient (rho).

The rho measures the linear dependence between a pair of variables (the time series x and y, which represent the neural activity in different brain regions). It c as.

(13) $$\rho =\frac{S_{xy}}{\sigma_{x}\sigma_{y}}$$

where *S* is the covariance and σ the standard deviation (Lee Rodgers and Nicewander, 1988; Wang et al., 2014). The rho can take values between − 1 and 1, where these values indicate perfect negative and positive linear relationships between x and y, respectively, whereas a value of 0 indicates no linear relationship.

##### 2.4.2.2. Mutual information (MI).

The MI measures the amount of information shared by two random variables (time series x and y). In other words, it is the reduction in the uncertainty of one random variable due to the knowledge of the other (Cover and Thomas, 2005). The MI can capture nonlinear and nonmonotonic dependencies. It can be expressed as.

(14) MI(X;Y)=H(X)+H(Y)−H(X,Y)

where H(X), and H(Y) are the Shannon’s entropy of x and y, respectively, whereas H(X;Y) represents Shannon’s joint entropy of $\left(x,y\right).$

The MI is a non-negative measure, whose upper bound is given by min{H(X),H(Y)}, and takes value zero when variables are statistically independent. Here, MI was computed using the Gaussian copulas approximation (Ince et al., 2017), an efficient computation framework that combines the statistical theory of copulas with the closed-form solution for the entropy multivariate Gaussian distributions. The Gaussian copula approximation was also used for the computation of the information-theoretic metrics described below.

##### 2.4.2.3. Conditional mutual information (CMI).

The CMI measures the amount of information shared between two random variables (time series, x and y, when a third variable (z) is given. In other words, the CMI is the reduction in the uncertainty of one random variable due to the knowledge of the other, when a third variable is given (Cover and Thomas, 2005). CMI can be expressed as.


(15) CMI(X;Y∣Z)=H(X∣Z)−H(X∣Y,Z)

Since Z can be a multivariate set, it can be interpreted as the activity of all brain regions except for X and Y. Consistently with the computation of MI, CMI was obtained using the Gaussian copulas approximation (Ince et al., 2017).

##### 2.4.2.4. Weighted mutual information (WMI).

The WMI is a weighted version of the MI, where MI is weighted by the Hamming distance between discretized representations of the variables. Discretization is achieved by transforming the signals into a sequence of symbols (King et al., 2013; Staniek and Lehnertz, 2008). Following the procedure described by Moguilner et al. (2018), the symbolic transformation considers the adjacent neighboring values of each time series to provide symbols as output (i.e., a symbol for the local increase of the signal, and the symbol b for the local decrease of the signal). In other words, the discretization transformed x(t) to the symbol ‘a’ if x(t−1)<x(t)<x(t+1), and to ‘b’ if x(t−1)>x(t)>x(t+1), and 0 otherwise. The symbolic weights can take values ranging from 0 to 1, indicating minimal and maximal similarity, respectively. It represents the minimum number of substitutions needed to modify one symbolic string to match the other by computing the Hamming distance between streams. The *WMI* can be presented as.

(16) $$WMI(X;Y)=sw(\hat{X},\hat{Y})MI(X;Y)$$

where $\hat{X}$, and $\hat{Y}$ are the symbolic reNpresentations of the time series X and Y, respectively.

##### 2.4.2.5. Organizational information (O_info).

It is a multivariate extension of Shannon’s mutual information (Cover and Thomas, 2005), which informs about the dominant characteristic of multivariate systems (i.e., high order interactions). The O_info (Ω) captures redundancy-dominated scenarios where three or more variables have copies of the same information, and synergy-dominated systems in which high-order patterns cannot be traced from low-order marginals. It can be expressed as.


(17) $$\Omega \left(X^{n}\right)=(n-2)H\left(X^{n}\right)+\sum_{j=1}^{n}\left[H\left(X_{j}\right)-H\left(X_{-j}^{n}\right)\right]$$

For the analysis of brain activity, z can be considered a multivariate time series representing the activity of all brain regions except for x and y. Therefore, O_info measures how synergistic or redundant is the relationship between two brain regions with respect to the rest of the regions.

### 2.5. Multi-feature analyses for dementia classification

We tested the ability of joint analyses of connectivity to discriminate dementia subtypes (i.e., AD and bvFTD) from HCs using the following multi-feature analyses:

- Classifications based on a single frequency-domain metric (e.g., laggedCoh) that integrate information from all EEG frequency bands.
- Classifications based on the integration of complementary frequency-domain metrics (e.g., instCoh, laggedCoh, and totalCoh).
- Classification $-\alpha_{\omega }L_{\omega ,t}\leq L_{\omega ,t}^{shift}\leq \alpha_{\omega }L_{\omega ,\;t}s$ based on the integration of all the time-domain metrics presented in Table 2.

These multi-feature analyses are computationally expensive due to the substantial number of features used for the classification. For instance, this analysis includes 26,568 features (values of connectivity) when integration of complementary frequency-domain metrics is conducted (three connectivity metrics, eight frequency bands, and 3321 interactions, where interactions are represented in each triangle of the 82 × 82 connectivity matrices). Therefore, a feature selection process preceded the multi-feature classification. For each pair of regions, the functional connectivity computed with a given metric was compared between each dementia subtype and HCs, using two-tailed non-parametric permutation tests (α=0.05;5000 randomizations) (Manly, 2018). Results were corrected for multiple comparisons using the Benjamini and Hochberg FDR method (Benjamini and Hochberg, 1995). Pairwise connections with statistically significant differences between HCs and AD, and between HCs and bvFTD, were selected for the next steps of the analysis.

Noteworthy, unlike recent studies (Birba et al., 2022; Herzog et al., 2022), a comparison between AD or bvFTD was not conducted. Comparing dementia subtypes would have required the implementation of patient-control normalizations based on *Z*-transformations of the data to rescue differences relative to HCs (Birba et al., 2022; Herzog et al., 2022). Considering the high computational cost of the multivariate machine learning classifications based on Z-transformed connectivity data, priority was given to the patient-controls analyses.

#### 2.5.1. Consistency score

A consistency score was computed for each pairwise connection. This score represents the number of metrics for which statistically significant difference were obtained in the AD - HCs contrast, or in the bvFTD - HCs contrast. The consistency score was obtained separately for both hypoconnectivity (reduced connectivity in dementia in comparison with HCs) and hyperconnectivity (increased connectivity in dementia in comparison with HCs). The maximum consistency score was 101, which represents the total of types of connectivity being assessed. Based on consistency score, pairwise *t*-test (*p* < 0.005, Bonferroni corrected) were conducted to stablish whether the representativity of regions with atypical functional connectivity in dementia varied among brain lobes and dementia subtypes. Brain lobes were frontal (F), limbic (L), occipital (O), parietal (P), and temporal (P).

#### 2.5.2. Machine learning approach

The functional connections that survived the statistical test were used as features of machine learning classifiers to discriminate AD from HCs, and bvFTD from HCs. Under guidelines for machine learning partition (Dobbin and Simon, 2011), the datasets were split into 80% and 20% for training and testing, respectively, using random divisions to test for generalizability without using the testing dataset during the validation phase for out of k - folds (k=10) predictions. The 8:2 split ratio has resulted in robust cross-validation of neuroimaging studies (Lanka et al., 2020). Following best practices guidelines, a k-fold (k = 10) cross-validation for Bayesian hyper-parameter tuning was used in the training phase of each multi-feature analysis (Poldrack et al., 2019). The feature importance analysis technique was implemented to establish the set of features most relevant in each classification scheme. The eXtreme Gradient Boosting (XGBoost) classifier library (Chen and Guestrin, 2016) was the choice of gradient boosting machine (GBM). XGBoost has high accuracy and robustness (Feurer and Hutter, 2019) and provides parallel computation tree boosting, resulting in fast and accurate predictions which have proven to be successful in different fields (Zheng et al., 2017). Furthermore, this classifier also provides regularized boosting, which helps to reduce overfitting and provides more generalizable results.

Following guidelines for reporting machine learning results (Uddin et al., 2019), classification accuracy values (F1 score) were reported. This parameter conveys the balance between the precision (the ability to classify positive samples) and the recall (how many positive samples were correctly classified) of the classification. The F1 scores were accompanied by (i) the receiver operating characteristic (ROC) curve, and (ii) the SHapley Additive exPlanations (SHAP) of relevant pairwise interaction (Lundberg and Lee, 2017). SHAP values connect optimal credit allocation with local explanations using the Shapley value and help construct the set of parameters that provide a compressive explanation of the classification.

## 3. Results

### 3.1. Atypical connectivity in dementia involved 1/10 of all connections, captured by 1/4 of the connectivity metrics

We analyzed whether source-localized rsEEG connectivity varied between AD and HCs, and between bvFTD and HCs when brain interactions were assessed with different connectivity metrics. As expected, atypical connectivity in dementia was restricted to a relatively small set of functional connections (less than a tenth of all 3280 possible interactions) (Fig. 1B and S1). Atypical connectivity in dementia, considering any brain interaction, was never captured by more than a quarter of all 101-connectivity metrics. (Fig. 1B).

#### 3.1.1. Temporo-posterior hypoconnectivity characterizes AD

Hypoconnectivity captured by at least one connectivity metric in AD (320 connections) accounted for almost thrice the number of hyperconnectivity in the same group of patients (121 connections) (Fig. 1B, left panels). On average (mean ± standard deviation), hypo and hyperconnectivity of a given connection in AD were captured by 3.7 ± 2.7, and 1.9 ± 1.0 connectivity measures, respectively. Consequently, in this group of patients, hypoconnectivity was more consistently observed than hyperconnectivity across metrics (t=5.13,p<0.001; Cohen’s d=0.65).

Among these atypical connections, the left precuneus - left calcarine sulcus (parieto- occipital network) was the AD hypoconnectivity that was captured by the largest set of connectivity measures (Fig. 1B, upper left panel). Nevertheless, even in this case, hypoconnectivity was captured by less than one-third (26) of all types of functional interactions (101). Both intra-parietal (supramarginal gyrus - precuneus) and parieto-limbic hypoconnectivity (involving the angular gyrus, hippocampus, and middle/posterior cingulate cortices) were also consistently observed (Fig. 1B).

AD hyperconnectivity with the highest consistency scores were restricted to frontotemporal areas, i.e., between medial and inferior frontal gyri, and between the inferior frontal gyrus and the superior temporal cortex (Fig. 1B and S1).

#### 3.1.2. Fronto-temporo-parietal hypoconnectivity and frontotemporal hyperconnectivity typifies bvFTD

Unlike AD, the number of hypoconnectivity captured by at least one connectivity measure in bvFTD (267 pairs), was lower than that of hyperconnectivity (307). Nevertheless, as with AD, the mean number of metrics that detected a given hypoconnectivity in bvFTD (3.0 ± 1.3) was statistically significantly higher than that detecting hyperconnectivity (1.8 ± 0.9, t=6.28,p<0.001; Cohen’s d=0.51).

Bilateral intra-hemispheric hypoconnectivity between the precentral gyrus and the temporal pole was the atypical connectivity captured by the largest set of connectivity measures in bvFTD (Fig. 1B). Decreased intra-hemispheric and inter-hemispheric frontal connectivity, as well as bilateral parieto-temporal hypoconnectivity (between the postcentral lobe and the temporal pole) were also captured by relatively large sets of connectivity measures (Fig. 1B and S1).

The bvFTD hyperconnectivity was mainly reflected by homotopic connections between the temporal poles, and by frontotemporal interactions, i.e., middle frontal gyrus - temporal pole, and orbital cortex - supramarginal gyrus (Fig. 1B). Furthermore, hyperconnectivity between limbic (amygdala, and insula) and temporal areas (temporal pole), as well as within the frontal lobe, were reflected by different types of functional interaction (Fig. 1B).

#### 3.1.3. Confirmation of the spatial distribution of atypical connectivity in dementia via consistency scores

A conjoint analysis of connectivity metrics provided a topographical representation of regions with atypical connectivity in each dementia subtype (Fig. 1C). Additional information on these conjoint analyses is presented in Fig. S2 and Table S2. Regions with the highest consistency score of hypoconnectivity in AD belonged to the temporal, parietal and occipital lobes (Fig. 1C). In a descending order, regions with highest consistency score of hyperconnectivity were the precuneus (bilaterally), the right paracentral lobe, the left angular gyrus, the right middle cingulum and the left calcarine (Fig. 1C). Conversely, areas in bvFTD with a high consistency score of hypoconnectivity were condensed in fronto-temporo-parietal locations. In this group, areas ranked highest in term of consistency score were the pre- and postcentral gyri (bilaterally), and the superior temporal pole (bilaterally) (Fig. 1C, Table S2).

Functional hyperconnectivity in both dementia subtype was most systematically observed in fronto-temporal regions (Fig. 1C). In AD, the areas with the highest consistency score of hyperconnectivity were the inferior frontal gyrus (triangular part, bilaterally), the left inferior frontal gyrus (opercular part), the left superior temporal pole and the left insula (Fig. 1C, Table S2). Areas with the highest consistency score of hyperconnectivity in bvFTD were the middle temporal pole (right), the right inferior frontal gyrus (triangular and opercular parts), the left inferior frontal gyrus (orbital region) and the left middle frontal gyrus (Fig. 1C, Table S2). Comparing the global consistency scores between dementia subtypes, higher scores of hypoconnectivity were obtained in AD (t=8.56,p<0.001 Bonferroni corrected). The opposite was observed for hyperconnectivity, which was most representative of bvFTD (t=9.66,p<0.001 Bonferroni corrected) (Fig. S2).

### 3.2. Dementia classification based on metric’s integration

To test the hypothesis that the integration of source-space EEG connectivity representations obtained with different connectivity metrics provides robust information to accurately predict dementia, machine learning classifiers were implemented. Results of the classifications are summarized in Table 3 (classifications based on a single frequency-domain connectivity metric that integrate information from all EEG frequency bands), Table 4 (classifications based on the integration of complementary frequency-domain metrics), and Table 5 (classifications based on the integration of time-domain connectivity metrics).

#### 3.2.1. Feature selection in the validation stage

**Fig. 2.**
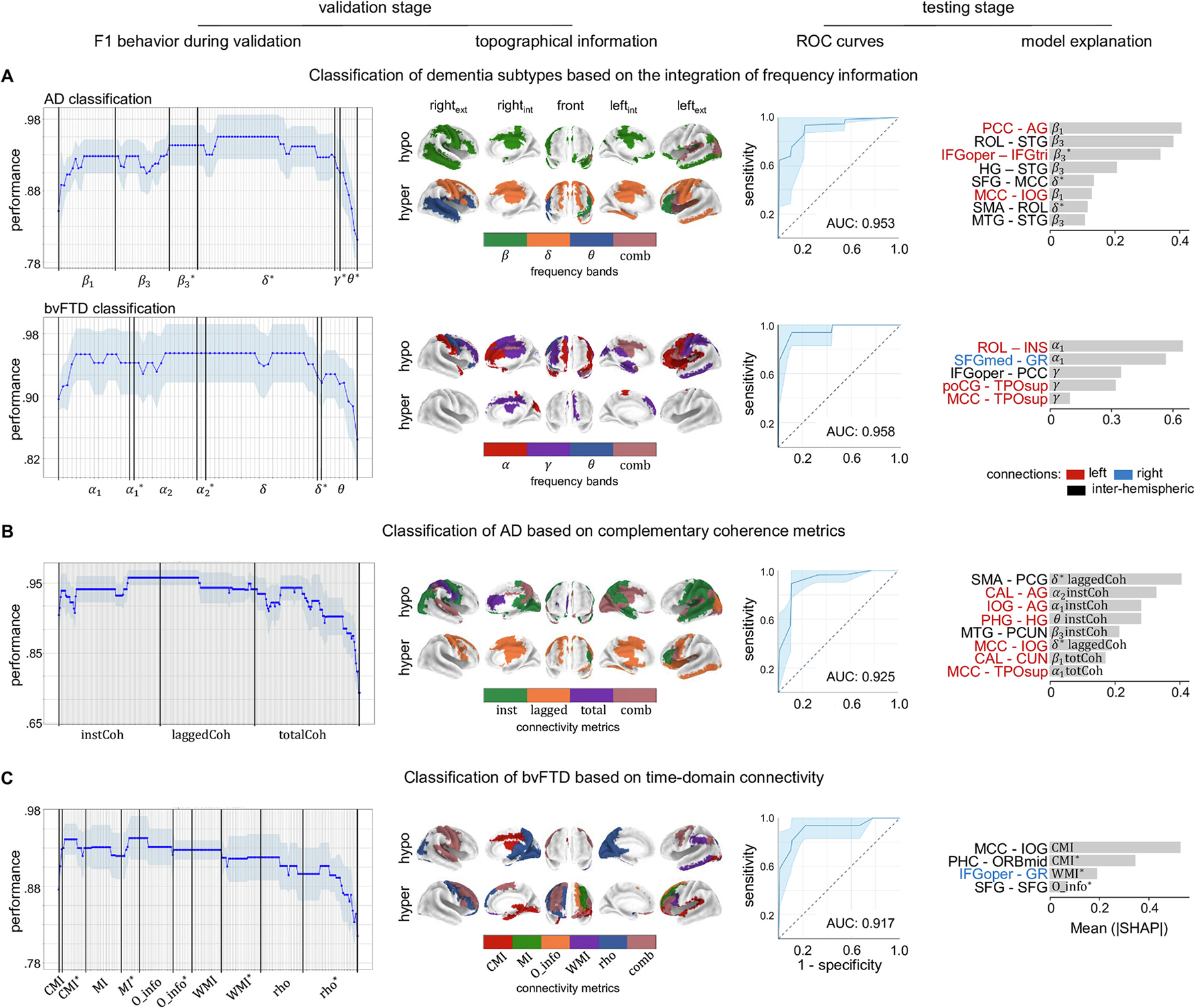
Examples of dementia classification based on source-localized rsEEG connectivity. A) Classification of Alzheimer’s disease (AD) based on functional connectivity estimated with lagged coherence (laggedCoh), and classification of behavioral variant frontotemporal dementia (bvFTD) when functional connectivity was estimated with total nonlinear connectivity (totalnLC). Since functional connectivity was estimated in each EEG frequency band, classification relied on the integration of based frequency information. B) Classification of AD based on the integration of complementary coherence-related connectivity metrics, which also integrated information from multiple EEG frequency bands. Metrics were instantaneous (instCoh), lagged (laggedCoh) and total (totalCoh) coherence. C) Classification of bvFTD based on the integration of different time-domain connectivity measures. For each classification, the performance of the classification (F1 during validation) is presented as a function of the number of features (functional connectivity) that were sequentially included in the analyses. The frequency bands and the types of functional connectivity are represented as appropriate. The * denotes features that represent hyperconnectivity. Otherwise, features represent hypoconnectivity. Extended names of the metrics are presented in Table 2. The topographical information of regions comprising the set of features selected for classification is illustrated in the panel topographical information. Regions denoted by ‘comb’ in the color-bar belong to atypical connections captured by different connectivity measures. The receiver operating characteristic curves (ROC curves) are presented, and the area under the curve (AUC) is noted for each case. ROC are presented as the mean (thick line) ± 95% confidence interval (shadows), which were obtaining by bootstrapping (5000 times). Connections wight greatest contribution to the predictive model are presented (model explanation). Intra and inter-hemispheric connections are colored differently. Extended names of the brain regions are presented in Table S1.

Representative examples of dementia classifications based on joint analyses of connectivity (Tables 3–5) are depicted in Fig. 2. The selection considered the different hypotheses tested with the machine learning analyses (Section 2.5.2). Therefore, two classifications based on a single frequency-domain connectivity metric integrating information from all EEG frequency bands are presented, one for each dementia subtype (Fig. 2A and B). Furthermore, an example of the classifications based on the integration of complementary frequency-domain metrics, and an example of the classifications based on the integration of time-domain connectivity metrics are also provided (Fig. 2C and D). Noteworthy, the classifications exemplified in Fig. 2 are not necessarily those with the highest performance (e.g., Fig. 2A and B), but show the benefits of the metrics integration proposed in this study.

During the validation stage, the performance of the classification (F1) systematically increased as more features (atypical connectivity) were subsequently incorporated (Fig. 2, F1 behavior during validation). As expected, after reaching the maximum F1, this score remained constant as additional features were incorporated, and decreased thereafter (Fig. 2, F1 behavior during validation).

The relevance of metrics integration for dementia prediction was evidenced in the set of parameters (optimum features) obtained during the validation stage (Fig. 2, topographical information), which were subsequently used for testing the classification models (Fig. 2, testing stage). This was the case of the classifications based on a single connectivity metric, which always conveyed information from different EEG frequency bands (Fig. 2A, topographical information; Table 3). Broader and more complex sets of selected features were obtained when either complementary frequency-domain (Fig. 2B, topographical information; Table 4) or time-domain connectivity metrics (Fig. 2C, topographical information; Table 5) were integrated. In fact, the optimum set of features for bvFTD/HCs classification based on time-domain connectivity metrics comprised connections estimated with five of the seven measures included in the analysis (CMI, MI, O_info, WMI and rho) (Fig. 2C, topographical information). Noteworthy, most of the set of optimum features for classifications comprised both hypo and hyperconnectivity (Fig. 2, topographical information; Tables 3–5).

#### 3.2.2. Classifications based on representations of functional connectivity in different EEG frequency bands assessed with a single connectivity metric

When the AD/HCs classifications relied on information gathered by a single connectivity metric in different EEG frequency bands (Table 3), the highest and lowest classification scores were obtained with instCoh and laggedPS, respectively. For bvFTD/HCs classifications, the highest and lowest performances were obtained with instCoh, and totalPS (Table 3), respectively. The set of strongest features for classification displayed a marked frequency-band specificity. The top features describing AD hypoconnectivity (AD connectivity < HCs connectivity) were obtained in the α and β frequency bands. For the bvFTD/HCs classification, this group of features comprised interactions in the α and γ bands (Table 3). In bvFTD, β hypoconnectivity was barely noticed (bvFTD/HCs classification). The most relevant hyperconnectivity (connections with highest SHAP scores) in both dementia subtypes were mainly obtained in the δ and θ bands. This was accompanied by α hyperconnectivity in bvFTD.

##### 3.2.2.1. Examples of classification based on information gathered by a single connectivity metric in different EEG frequency bands.

In the examples illustrated in Fig. 2A, the atypical connections captured by laggedCoh that better explained the AD/HCs classification (connections with the highest SHAP scores) were restricted to the β1,β3 and δ EEG frequency bands, and represented both hypo- (in β1 and β3) and hyperconnectivity (β3 and δ) (Fig. 2A, model explanation). These top features for classification included hypoconnectivity between the (a) cingulate cortex (middle and posterior cingulate cortex) and the superior frontal gyrus, (b) inter-hemispheric fronto- temporal connections (Rolandic operculum and the superior temporal gyrus), as well as (c) inter-hemispheric temporal interactions. Furthermore, this AD/HCs classification was mostly explained by hyperconnectivity between (a) the middle cingulate and superior frontal cortices, as well as (b) connections restricted to the frontal lobe (involving the operculum and the supplementary motor area; Fig. 2A, model explanation).

The top features of the bvFTD/HCs classification based on totalnLC (Fig. 2A) exclusively represented α1 and γ hypoconnectivity (Fig. 2A, model explanation). These connections involved frontal areas (gyrus rectus, operculum, inferior frontal gyrus, and medial superior frontal gyrus), as well as limbic (insula, and cingulate cortex), temporal (temporal pole) and parietal (postcentral gyrus) cortices. In this case, the group of features with highest SHAP scores did not include connections with increased connectivity in bvFTD relative to HCs (Fig. 2A, model explanation).

#### 3.2.3. Classifications based on the integration of complementary frequency-domain connectivity metrics

The most robust and accurate AD/HCs classification based on the integration of complementary frequency-domain connectivity metrics were obtained with coherence- related measures (instCoh, laggedCoh and totalCoh). For equivalent sets of metrics, the highest bvFTD/HCs classification was obtained with complementary linear connectivity (instLC, laggedLC and totaLC), (Table 4). The lowest classification scores resulted from integrating nonlinear connectivity (instnLC, laggednLC and totalnLC), and phase synchronization measures (instPS, laggedPS and totalPS), for the AD/HCs and the bvFTD/classifications respectively.

As expected, the top features that resulted from joint analyses of frequency-domain connectivity metrics already displayed the frequency-specific atypical connectivity described in Section 3.2.2. Without exception, both AD and bvFTD hyperconnectivity (higher connectivity compared to HCs) were exclusively captured by lagged connectivity measures. Furthermore, zero-lag connections were consistently associated with hypoconnectivity (Table 4). Most of these classifications relied on both instantaneous and lagged connectivity. The exceptions were the AD classification, which was based on nLC, and the bvFTD classification based on PS, for which none of the top features for classification involved instantaneous and lagged atypical connectivity, respectively (Table 4). These measures (nLC and PS) provided the lowest classification of AD/HCs and bvFTD/HCs, respectively. Although classifications based on LC did not rely on totalLC, it is of note that this metric is the sum of instLC and laggedLC, and that this latter connectivity metric already captured atypical connectivity crucial for classification (Table 4).

**Fig. 3.**
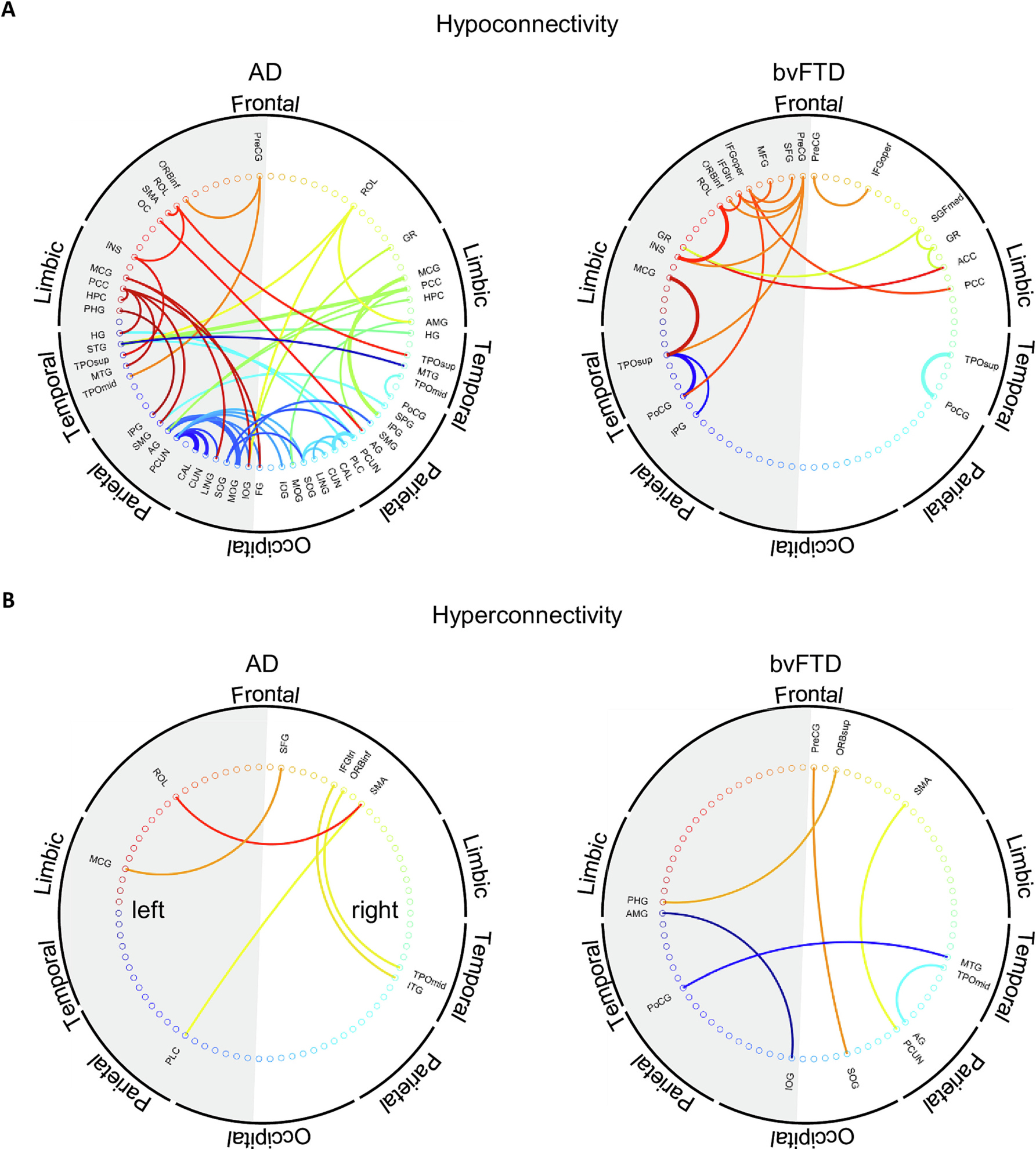
Connections contributing to the classification of Alzheimer’s disease (AD) and behavioral variant frontotemporal dementia (bvFTD) relative to elderly healthy controls (HCs), when machine learning classifications were based on the integration of complementary frequency-domain connectivity metric. A) Topographic patterns of hypoconnectivity. B) Topographic patters of hyperconnectivity. Different lobes are indicated around the circles. Different connections are presented with different colors. The grey half circle denotes the left cerebral hemisphere. In each plot, the line width represents the relative contribution of the connections to the classifications, i.e., the number of joint analyses of connectivity for which a connection contributed to discriminate a dementia subtype from HCs, relative to the total number of joint analyses of connectivity.

The joint analysis of the connections with the highest influence in classifications that integrated complementary frequency-domain connectivity metrics illustrate that these classifications primarily relied on hypoconnectivity (Fig. 3A), although they were also determined by hyperconnectivity (Fig. 3B). The topographic distribution of hypoconnectivity was specific to each dementia subtype. Relevant hypoconnectivity for AD/HCs classification mainly comprised intra-hemispheric connections, involving temporal, parietal, and occipital regions (Fig. 3A, left panel). In the bvFTD/HCs classifications, relevant hypoconnectivity was concentrated in rostral regions (Fig. 3A, right panel). Critical hyperconnectivity in both conditions displayed a wide distribution, involving different regions in the frontal, limbic, temporal, parietal and occipital lobes (Fig. 3B).

##### 3.2.3.1. Example of classification based on complementary frequency-domain connectivity metrics (coherence related metrics).

Continuing the example illustrated in Fig. 2B the set of features that better explained the AD/HCs classification based on Coh-related metrics comprised hypoconnectivity between (a) middle cingulate - temporal pole, (b) parietal cortex (angular gyrus, precuneus) - superior temporal gyrus and occipital areas, as well as (c) hippocampal regions - temporal lobe (Fig. 2B, model explanation). Hyperconnectivity, in turn, involved the middle cingulate cortex, as well as frontal (precentral gyrus and supplementary motor area) and parietal (postcentral gyrus) areas (Fig. 2B, model explanation).

#### 3.2.4. Classifications based on the integration of time-domain connectivity metrics

Joint analyses of time-domain connectivity metrics resulted in slightly lower classification scores than the integration of frequency-domain connectivity metrics (Tables 4 and 5). Furthermore, these scored did not varied between the AD/HCs and the bvFTD classifications (Table 5). While the top features for the AD/HCs classification were only captured with CMI, the bvFTD/HCs classification relied on different time-domain metrics (Table 5). In both cases, the set of features that better explained this classification included hypo and hyperconnectivity.

##### 3.2.4.1. Example of classification based on time-domain connectivity metrics.

The final example of classification illustrated in Fig. 2 (bvFTD /HCs classification based on time-domain connectivity metrics) illustrated that the atypical connections with greatest contribution to the classification (connections with highest SHAP) were captured by CMI, WMI, and O_info (Fig. 2C). Three of the four connectivity with highest SHAP values informed about hyperconnectivity in bvFTD. Relevant connections involved frontal regions (orbital cortex, operculum, gyrus rectus, and superior frontal gyrus), the parahippocampal gyrus, and the cingulate cortex.

## 4. Discussion

Whole-brain functional connectivity was investigated in a well-haracterized cohort of AD and bvFTD patients. Analyses revealed that less than a tenth of all 3280 possible connections in the EEG source space were responsible for the differences between each dementia subtype and HCs (Fig. 1B). Atypical connectivity included both hipo- and hyperconnectivity, which were never captured by more than a quarter of all 101 possible types of functional interactions. These results highlight the complex and synergetic nature of brain-phenotype associations (Ibanez, 2022). Hypoconnectivity (decreased connectivity relative to HCs) was topographically distributed over temporo-posterior brain regions in AD, and over fronto-temporo-parietal locations in bvFTD (Fig. 1B and C). In both dementia subtypes, hyperconnectivity was restricted mainly to fronto-temporal areas (Fig. 1B and C). Atypical connectivity in dementia was frequency-band specific, and dementia classifications typically relied on the integration of different EEG frequency bands, and/or different connectivity metrics (Fig. 2, Tables 3–5). The joint analyses presented in this study offers a valid alternative for the selection problem in EEG connectivity, allowing the assessment of complex connectivity patterns that may be overlooked with single metric approaches.

### 4.1. Integration of functional connectivity metrics

#### 4.1.1. Contribution of joint analyses of functional connectivity

This study confirms the relevance of integrationist approaches for dementia characterization. Generally, dementia classifications that rely on functional connectivity outperform those based on spectral markers (Blinowska et al., 2017; Hughes et al., 2019). Likewise, dementia classifications based on the integration of functional connectivity measures offer more robust results than single metrics (Alonso et al., 2011; Blinowska et al., 2017). Although probably critical for EEG due to the nature and temporality of electric signals, benefits of joint analyses of functional connectivity have also been suggested in fMRI-based studies, where the use of a composite metric of functional connectivity has been proposed (Mohanty et al., 2020).

Joint analyses of different EEG frequency bands, and different connectivity metrics, have been proposed for dementia characterization (Alonso et al., 2011; Briels et al., 2020; Hughes et al., 2019). Our results support this framework and bring novel evidence regarding the integration of related connectivity metrics. For instance, a heterogeneous set of features in terms of spectral frequency bands (Fig. 2A, Tables 3 and 4) and connectivity metrics (Fig. 2B and C, Tables 4 and 5) provided the most accurate classification of AD and bvFTD. Only the AD classification based on time-domain connectivity metrics exclusively relied on one connectivity metric (i.e., CMI) (Table 5). The latter result was unexpected, and further analyses are needed to provide a plausible explanation for it.

#### 4.1.2. Assessing source space connectivity

Since the estimation of functional connectivity in the EEG/MEG source space is sensitive to the head volume conduction (Colclough et al., 2016), the use of metrics that discard zero lag interaction has been proposed (e.g., Babiloni et al., 2020; Gaubert et al., 2019; Schoonhoven et al., 2022). Nevertheless, a fair balance between having non-physiological spurious interactions and providing the highest possible spatial resolution is difficult to achieve in the EEG source space. Furthermore, the neglect of zero lag interactions may lead to underrepresenting genuine functional connections among nearby cortical loci (Schoonhoven et al., 2022). Therefore, the integration of metrics with different sensitivity to the head volume conduction (Fig. 2B, Table 4) represents a pragmatic approach to EEG connectivity. The validity of this alternative is illustrated by the fact that, except for two classifications, atypical connectivity that contributed most to the classifications based on frequency-domain complementary metrics always comprised both lagged and instantaneous interactions (Table 4).

### 4.2. Specific patterns of atypical connectivity characterize AD and bvFTD

#### 4.2.1. Hypoconnectivity in dementia

The frequency-band specificity of the AD hypoconnectivity (Tables 3 and 4) is in line with previous EEG/MEG studies using envelope correlation (Briels et al., 2020; Koelewijn et al., 2017; Núñez et al., 2019; Schoonhoven et al., 2022), and both coherence and synchronization related measures (e.g., Alonso et al., 2011; Babiloni et al., 2016; Schoonhoven et al., 2022). Likewise, the AD hypoconnectivity observed with CMI (Table 5) is consistent with results obtained with other MI-related metrics (Dauwels et al., 2010; Vyšata et al., 2015; Waser et al., 2016). Connectivity studies in bvFTD are relatively scarce, and results are less consistent (Livinț Popa et al., 2021). Nevertheless, the results presented in Fig. 2 and Table 5 confirm the decrease in phase lag index of α oscillations, and the absence of changes in β connectivity previously reported for this dementia subtype (Yu et al., 2016).

The fact that most connections that are crucial for dementia/HCs classifications were biased toward hypoconnectivity support previous findings in neurodegeneration (AD studies: Briels et al., 2020; Herzog et al., 2022, Pineda-Pardo et al., 2014; Stam, 2014; Vecchio et al., 2017; bvFTD studies: Filippi et al., 2013; Yu et al., 2016). The temporo-posterior hypoconnectivity observed in AD (Fig. 1C, Fig. S3, Table S2), included the posterior cingulate cortex, precuneus, and hippocampus, which represent relevant nodes of the default mode network (DMN) significantly affected in this condition (De Haan et al., 2012; Hafkemeijer et al., 2015; Zhou et al., 2010). Remarkably, the distribution of consistency scores (Fig. 1C) resembles the brain topography of AD hypoconnectivity described with MEG (Schoonhoven et al., 2022). Considering this spatial information and the role of the DMN in episodic memory retrieval, mental state attribution, and visual imagery (Zhou and Seeley, 2014), our study is in line with previous findings demonstrating that AD hypoconnectivity is mainly observed in posterior brain regions (Engels et al., 2017; Li et al., 2015), whose weakened connectivity has been associated with decreased cognitive performance (Engels et al., 2017; De Haan et al., 2012; Teipel et al., 2016).

The fronto-temporo-parietal hypoconnectivity in bvFTD involved the cingulum, the postcentral, supramarginal and superior temporal gyri, as well as the insula (Fig. 1C, Table S1). These areas match regions with severe atrophy in bvFTD (Ibañez and Manes, 2012; Ibáñez et al., 2017; Ibáñez, 2018; Migeot et al., 2022; Birba et al., 2022; O’Connor et al., 2017) and represent hubs of the salience and the auditory networks impaired in this condition (Hafkemeijer et al., 2015; Seeley, 2019; Zhou et al., 2010) (Fig. S3). Other areas with high consistency scores of hypoconnectivity were the dorsolateral prefrontal and lateral orbitofrontal cortices, also impaired in bvFTD (Migeot et al., 2022; Seeley, 2019; Zamboni et al., 2008).

Hypoconnectivity in dementia can be partially explained by brain atrophy. In fact, the grey volume density of the occipital lobe of AD patients has been associated with the activity of the neural generators of α oscillations (Babiloni et al., 2016). Atrophy is accompanied by disintegration of both subcortical and cortical tracts. Nevertheless, the frequency-band specificity of hypoconnectivity indicates that this process also represents the hampered ability of the remaining neurons to coordinate neural activity (Uhlhaas and Singer, 2006; Núñez et al., 2019). This impaired neuronal coupling may result from the disruption of thalamo-cortical and cortico-cortical communication systems responsible for generating ongoing brain rhythmicity (Babiloni et al., 2016; Núñez et al., 2019), in which cholinergic, GABAergic and glutamatergic neurotransmissions are compromised.

#### 4.2.2. Hyperconnectivity in dementia

Although less consistently observed, previous studies have also reported AD hyperconnectivity in the δ and θ frequency bands of the EEG/MEG (Herzog et al., 2022; Alonso et al., 2011; Briels et al., 2020; Hsiao et al., 2013; Ruiz-Gómez et al., 2019; Schoonhoven et al., 2022). Likewise, δ and α hyperconnectivity have been observed in bvFTD (Yu et al., 2016). These findings, along with novel results presented here (Tables 3 and 4; Fig. S3) indicate that, hyperconnectivity in dementia is also frequency-band specific, and that hypo- and hyperconnectivity in neurodegeneration are visible in different frequency ranges (Engels et al., 2017).

The divergent connectivity pattern of the DMN and the SN in AD and bvFTD, in which the network with the greatest hypoconnectivity in one of the diseases displays the greatest hyperconnectivity in the other (Migeot et al., 2022; Greicius et al., 2004; Zhou et al., 2010, Zhou and Seeley, 2014), was only partially replicated in our study. Nevertheless, AD hyperconnectivity was consistently observed in relevant nodes of the SN (Zhou et al., 2010), including the inferior frontal gyrus, the insula, the temporal pole, the amygdala, and the hippocampus (Fig. 2C and S3, Table S2). By contrast, bvFTD hyperconnectivity was consistently observed in frontal nodes of the DMN (Greicius et al., 2004), e.g., the dorsolateral and medial prefrontal cortices, as well as the orbitofrontal cortex (Fig. 2C and S3). Likewise, regions with a high consistency score of hyperconnectivity in the right hemisphere of bvFTD (inferior frontal gyrus, middle frontal gyrus, amygdala, and middle temporal gyrus) matched those associated with specific symptoms of this dementia subtype (O’Connor et al., 2017; Zamboni et al., 2008).

Our results indicate that hyperconnectivity, besides representing a compensatory mechanism in preclinical stages (Devos et al., 2022;), is present in the middle stage of the disease. In this stage, hyperconnectivity in neurodegeneration might be explained by a decrease in the dynamic range of cortical neurons associated with reduced neurite length, shifts in the site of generation of action potentials toward central portions of the neuron, and impaired inhibitory interneuron and glial function (Dubey et al., 2022; Ghatak et al., 2019; Kazim et al., 2021; Targa Dias Anastacio et al., 2022).

### 4.3. Limitations and future directions

The main limitations of this study are i) the use of a small-to-moderate sample size, ii) the lack of control for demographic covariates (age, sex and ages of formal education) (Maito et al., 2023; Moguilner et al., 2022; Santamaría-García et al., 2021), iii) the absence of external datasets for the out of sample validations of the classification models (Schoonhoven et al., 2022), iv) the use of a single EEG source localization method, and v) the non-inclusion of directed functional connectivity metrics such as Granger causality and the directed transfer function (Cassani et al., 2018). Furthermore, the information redundancy provided by different connectivity metrics (Dauwels et al., 2010) was not assessed in our study. Less computationally expensive algorithms, and better classification performances, may prove worthy if integration is restricted to uncorrelated types of functional connectivity. Likewise, the potential benefits of integrating frequency- and time-domain connectivity metrics need to be further assessed. Finally, reproducibility studies need to include other methods to solve the inverse problem of the EEG/MEG and address the strategy to compute the time series from which functional connectivity is estimated. While the average of voxels belonging to the same region of interest has been implemented in this and other studies (Gaubert et al., 2019), time series have been extracted with principal component analyses and by choosing the nearest voxel to the centroid of the area (Hughes et al., 2019).

## 5. Conclusions

Disparate methodological approaches to functional connectivity have been used in dementia research, with no consensus on the best approach. In this study, the sets of functional connectivity with greatest contribution to the classification of different dementia subtypes are heterogeneous, comprising interactions in different EEG frequency bands and/or interactions captured by different connectivity measures. The information gathered from these joint analyses of connectivity offers a comprehensive description of whole-brain functional interactions and provides a thorough set of features to accurately classify dementias.

Future studies need to address the construction of a less complex description of this composite multi-metric representation of functional connectivity, e.g., by collapsing all the information into a single connectivity matrix. This refinement, along with the implementation of classifiers that combine spectral and connectivity descriptors of the EEG, will certainly contribute to developing potential clinical tools for assessing neurodegeneration.

## Supplementary Material

1

## Figures and Tables

**Table 1:** Demographic information and neuropsychological performance.

	bvFTD (*N* = 19)	AD (N = 33)	HCs (N = 42)	Statistic (All groups)	Post-hoc comparisons	
					Contrast	*p*-value

Demography						
Sex (F:M)	5:14	18:11	22:9	χ^2^ = 10.64 *p* = 0.006^a^	HCs-AD HCs-bvFTD	0.31^b^ 0.08^b^
Age (years)	68.57 (1.92)	74.65 (1.55)	69.87 (1.50)	F = 2.13 *p* = 0.08^a^	HCs-AD HCs-bvFTD	0.70^c^ 0.85^c^
Education (years)	14.57 (0.91)	11.20 (0.74)	13.64 (0.71)	F = 2.13 p = 0.08^a^	HCs-AD HCs-bvFTD	0.60c 0.70c
Neurophysiological assessment						
Cognitive state (MoCa)	22.22 (0.92)	16.48 (0.72)	25.66 (0.75)	F = 16.12 p < 0.001^a^	HCs-AD HCs-bvFTD	*<*0.001^c^ 0.01
Executive functions (IFS)	19.66 (0.90)	14.43 (0.71)	23.45 (0.78)	F = 36.99 p < 0.001^a^	HCs-AD HCs-bvFTD	*<*0.001^c^ 0.01
Facial emotion recognition	10.16 (2.63)	9.78 (2.83)	12.35 (1.80)	F = 8.74 *p* = 0.001^a^	HCs-AD HCs-bvFTD	*<*0.001^c^ 0.009

Data are presented as mean (SD), except for sex. Superscripts represent the statistical test to which the of p-values belong:

a one-way ANOVA,

b chi-squared test (χ^2^),

c Tuckey HSD test. bvFTD: behavioral variant frontotemporal dementia, AD: Alzheimer’s disease, HCs: elderly healthy controls, MoCA: Montreal Cognitive Assessment, IFS: Ineco Frontal Screening.

**Table 2:** Metrics selected for the analysis of whole-brain functional connectivity.

Functional connectivity		

	Frequency-domain	Time-domain

Linear metrics	Linear connectivity (LC) Coherence (Coh)	Pearson's correlation (rho)
Nonlinear metrics	Nonlinear connectivity (nLC) Phase synchronization (PS)	Mutual information (MI) Weighted mutual information (WMI) Conditional mutual information (CMI) O-information (O_info)

**Table 3:** Classification of dementia subtypes based on joint analyses of functional connectivity estimated in different EEG frequency bands with a single connectivity metrics.

Connectivity metric	Type of connectivity	Classification	F1 score	Accuracy	Sensitivity	Specificity	Precision	Recall	EEG frequency band of relevant connections

Coherence	instantaneous	AD	0.982 (0.002)	0.983 (0.002)	0.983 (0.001)	0.990 (0.005)	0.983 (0.001)	0.983 (0.005)	*α*_1_, *α*_2_, *β*_1_, *β*_2_, *β*_3_
		bvFTD	0.993 (0.003)	0.975 (0.003)	0.990 (0.006)	0.984 (0.002)	0.988 (0.002)	0.976 (0.005)	*α*_1_, *α*_2_,
	lagged	AD	0.953 (0.006)	0.966 (0.004)	0.968 (0.002)	0.956 (0.001)	0.956 (0.006)	0.964 (0.001)	*β*_1_, *β*_3_, *β*_3_*, δ*
		bvFTD	0.960 (0.005)	0.968 (0.005)	0.965 (0.001)	0.955 (0.006)	0.967 (0.003)	0.952 (0.002)	*α*_2_*, δ*
	total	AD	0.929 (0.002)	0.934 (0.005)	0.934 (0.003)	0.941 (0.003)	0.946 (0.002)	0.938 (0.001)	*α*_2_, *β*_1_, *β*_3_
		bvFTD	0.928 (0.002)	0.930 (0.006)	0.933 (0.005)	0.942 (0.002)	0.946 (0.005)	0.943 (0.003)	*α*_1_, *γ*
Phase coherence	instantaneous	AD	0.908 (0.005)	0.907 (0.002)	0.906 (0.002)	0.906 (0.006)	0.890 (0.005)	0.904 (0.006)	*α*_1_, *α*_2_, *β*_3_
		bvFTD	0.894 (0.003)	0.907 (0.006)	0.909 (0.002)	0.901 (0.005)	0.907 (0.003)	0.909 (0.003)	*δ*, *γ*, *θ*
	lagged	AD	0.884 (0.004)	0.881 (0.004)	0.878 (0.006)	0.889 (0.002)	0.881 (0.003)	0.878 (0.006)	*β*_1_, γ*, θ*,
		bvFTD	0.929 (0.004)	0.938 (0.006)	0.925 (0.005)	0.930 (0.003)	0.944 (0.004)	0.938 (0.006)	*α*_2_*, *β*_1_, θ*
	total	AD	0.938 (0.002)	0.931 (0.003)	0.945 (0.005)	0.933 (0.004)	0.940 (0.002)	0.939 (0.006)	*β*_2_
		bvFTD	0.929 (0.004)	0.938 (0.006)	0.925 (0.005)	0.930 (0.003)	0.944 (0.004)	0.938 (0.006)	*α*_1_, *γ*
Linear connectivity	instantaneous	AD	0.981 (0.006)	0.974 (0.004)	0.987 (0.002)	0.974 (0.002)	0.976 (0.001)	0.993 (0.006)	*α*_1_, *β*_1_, *β*_2_, *β*_3_
		bvFTD	0.995 (0.002)	0.98 (0.002)	0.979 (0.006)	0.982 (0.002)	0.989 (0.006)	0.983 (0.002)	*α*_2_, *γ*
	lagged	AD	0.950 (0.006)	0.963 (0.004)	0.949 (0.005)	0.691 (0.005)	0.944 (0.002)	0.957 (0.002)	*β*_1_, δ*
		bvFTD	0.963 (0.005)	0.945 (0.005)	0.950 (0.001)	0.958 (0.005)	0.959 (0.006)	0.960 (0.004)	*α*_2_*, δ*
	total	AD	0.943 (0.002)	0.947 (0.001)	0.956 (0.001)	0.941 (0.006)	0.946 (0.006)	0.945 (0.006)	*α*_1_, *α*_2_, *β*_1_, *β*_3_
		bvFTD	0.959 (0.006)	0.946 (0.006)	0.954 (0.006)	0.947 (0.003)	0.949 (0.003)	0.951 (0.006)	*α*_1_, *α*_2_
	instantaneous	AD	0.932 (0.001)	0.944 (0.005)	0.936 (0.006)	0.929 (0.006)	0.937 (0.006)	0.927 (0.004)	*β*_2_, *γ*
		bvFTD	0.927 (0.006)	0.939 (0.003)	0.940 (0.006)	0.936 (0.005)	0.934 (0.005)	0.943 (0.03)	*α*_1_, *θ*
Nonlinear connectivity	lagged	AD	0.892 (0.005)	0.894 (0.006)	0.900 (0.002)	0.905 (0.006)	0.899 (0.002)	0.987 (0.006)	*β*_1_, *β*_2_, θ*
		bvFTD	0.901 (0.002)	0.896 (0.002)	0.905 (0.003)	0.901 (0.006)	0.895 (0.004)	0.906 (0.006)	*α*_1_, *β*_3_*, δ*
	total	AD	0.937 (0.004)	0.936 (0.006)	0.936 (0.005)	0.930 (0.006)	0.941 (0.003)	0.948 (0.006)	*α*_1_, *β*_1_, *β*_2_, *β*_3_, *γ*
		bvFTD	0.932 (0.006)	0.932 (0.006)	0.939 (0.002)	0.949 (0.003)	0.941 (0.006)	0.939 (0.003)	*α*_1_, *γ*

Results are presented as mean (95%confidence interval). The * denotes frequency bands at which functional connectivity in dementia was increased relative to controls. AD: Alzheimer’s disease, bvFTD: behavioral variant fronto-temporal dementia.

**Table 4:** Dementia classification based on the integration of complementary frequency-domain connectivity metrics.

Connectivity metric	Classification	F1 score	Accuracy	Sensitivity	Specificity	Precision	Recall	frequency-band of the top features for classifications		
								instantaneous	lagged	total

coherence	AD	0.9931 (0.006)	0.926 (0.001)	0.936 (0.004)	0.929 (0.004)	0.939 (0.006)	0.930 (0.002)	*α*_1_, *α*_2_, *β*_1_, *β*_2_, *β*_3_, *θ*	*β*_1_, *β*_3_, *β*_3_*, *θ*, δ*	*α*_1_, *α*_2_, *β*_2_, *β*_3_
	bvFTD	0.927 (0.003)	0.935 (0.002)	0.945 (0.006)	0.939 (0.004)	0.925 (0.003)	0.937 (0.004)	*α*_1_, *α*_2_, *γ*	*α*_2_ *	*α*_1_
phase synchronization	AD	0.9825 (0.003)	0.829 (0.002)	0.823 (0.001)	0.827 (0001)	0.831 (0.003)	0.823 (0.004)	*α*_1_, *α*_1_, *β*_1_, *β*_2_	-	*β*_3_, *γ*
	bvFTD	0.831 (0.006)	0.826 (0.003)	0.829 (0.003)	0.823 (0.002)	0.831 (0.001)	0.835 (0.003)	*α*_1_, *α*_2_, *γ*,	*β*_2_*, θ*	*α*_1_, *α*_2_
linear connectivity	AD	0.995 (0.005)	0.980 (0.003)	0.995 (0.006)	0.978 (0.006)	0.992 (0.003)	0.989 (0.002)	*α*_1_, *α*_2_, *β*_1_, *β*_3_	*γ* *	^-^
	bvFTD	0.995 (0.002)	0.980 (0.002)	0.979 (0.006)	0.982 (0.006)	0.989 (0.002)	0.983 (0.002)	*α*_1_, *α*_2_, *γ*	*α*_2_*, δ*	-
nonlinear connectivity	AD	0.949 (0.006)	0 0.938 (0.002)	0.938 (0.001)	0.934 (0.006)	0.930 (0.001)	0.943 (0.001)	*α*_2_, *β*_2_, *γ*	*β*_1_, δ*	*β*_1_, *β*_3_
	bvFTD	0.930 (0.003)	0.949 (0.004)	0.939 (0.003)	0.939 (0.006)	0.937 (0.002)	0.944 (0.002)	-	*α*_1_, *α*_2_*β_2_*, δ*	*δ*

Results are presented as mean (95%confidence interval). The * denotes frequency bands at which functional connectivity in dementia was increased relative to controls. AD: Alzheimer’s disease’s, bvFTD: behavioral variant fronto-temporal dementia.

**Table 5:** Classification of dementia subtypes based on the integration of time-domain connectivity metrics.

Classification	Fi score	Accuracy	Sensitivity	Specificity	Precision	Recall	Connectivity metrics contributing to the classification

AD	0.865(0.004)	0.871(0.006)	0.876(0.006)	0.877(0.004)	0.869(0.003)	0.869(0.006)	CMI, CMI*
bvFTD	0.863(0.003)	0.867(0.003)	0.874(0.003)	0.867(0.003)	0.866(0.006)	0.822(0.006)	CMI, CMI*, WMI*, O_info*

Results are presented as mean (95%confidence interval). The * denotes metrics for which functional connectivity in dementia was increased relative to controls. AD: Alzheimer’s disease, bvFTD: behavioral variant fronto-temporal dementia.

## Data Availability

Raw data is available upon reasonable request. Functional connectivity matrices are available at https://figshare.com/projects/Functional_connectivity_in_dementia/160601

## Abbreviations:

AAL: automated anatomical labeling
AD: Alzheimer’s disease
bvFTD: behavioral variant frontotemporal dementia
Coh: coherence
CMI: Conditional mutual information
FDR: false discovery rate
HCs: elderly healthy individuals
hdEEG: high-density electroencephalography
ICA: Independent Component Analysis
instCoh: instantaneous coherence
instLC: instantaneous linear connectivity
instnLC: instantaneous nonlinear connectivity
instPS: instantaneous phase synchronization
laggedCoh: lagged coherence
laggedLC: lagged linear connectivity
LC: linear connectivity
laggednLC: lagged nonlinear connectivity
laggedPS: lagged phase synchronization
MI: mutual information
MRI: magnetic resonance imaging
MNI: Montreal Neurologic Institute
nLC: nonlinear connectivity
O_info: Organizational information
PS: hase synchronization
ReDLat: Multi-Partner Consortium to Expand Dementia Research in Latin America
rsEEG: resting estate EEG
rho: Pearson’s correlation coefficient
SHAP: SHapley Additive exPlanations
sLORETA: standardized Low-Resolution Electromagnetic Tomography
totalCoh: total coherence
WMI: weighted mutual information
