# Supplementary material for: Source space connectomics of neurodegeneration: One-metric approach does not fit all
Source: Neurobiol Dis. Author manuscript; Available in PMC 2024 Jun 13. (PMC11170467; doi:10.1016/j.nbd.2023.106047)
Supplement: 1 [file NIHMS1996711-supplement-1.docx]

## Supplementary information

##### Table S1

Table S1: List of brain regions used as seeds for the estimation of functional connectivity from source-localized rsEEG

| Lobule | Region | Abbreviation |
| --- | --- | --- |
| Frontal | Precentral | PreC |
|  | Frontal Superior | SFG |
|  | Frontal Superior (orbital) | ORBsupmed |
|  | Frontal Middle | MFG |
|  | Frontal Middle (orbital) | ORBmid |
|  | Frontal Inf (opercular) | IFGoper |
|  | Frontal Inferior (triangular) | IFGotri |
|  | Frontal Inferior (orbital) | ORBinf |
|  | Rolandic Operculum | ROL |
|  | Supplementary Motor Area | SMA |
|  | Olfactory Bulb | OC |
|  | Frontal Superior Medial | SGFmed |
|  | Frontal Medial (orbital) | ORBsupmed |
|  | Rectus | GR |
| Limbic | Insula | INS |
|  | Cingulum Anterior | ACC |
|  | Cingulum Middle | MCC |
|  | Cingulum Post | PCC |
|  | Hippocampus | HPC |
|  | ParaHippocampal | PHG |
|  | Amygdala | AMG |
| Occipital | Calcarine | CAL |
|  | Cuneus | CUN |
|  | Lingual | LING |
|  | Occipital Superior | SOG |
|  | Occipital Middle | MOG |
|  | Occipital Inferior | IOG |
|  | Fusiform | FG |
| Parietal | Postcentral | PoCG |
|  | Parietal Superior | SPG |
|  | Parietal Inferior | IPG |
|  | SupraMarginal | SMG |
|  | Angular | AG |
|  | Precuneus | PCUN |
|  | Paracentral Lobule | PLC |
| Temporal | Heschl | HG |
|  | Temporal Superior | STG |
|  | Temporal Pole (superior) | TPOsup |
|  | Temporal Middle | MTG |
|  | Temporal Pole (middle) | TPOmid |
|  | Temporal Inferior | ITG |

##### Table S2

**Table S2**: Consistency score of atypical connectivity in dementia

| **Alzheimer’s disease** | | | | | | **Behavioral variant frontotemporal dementia** | | | | | |
| --- | --- | --- | --- | --- | --- | --- | --- | --- | --- | --- | --- |
| **hypoconnectivity** | | | **hyperconnectivity** | | | **hypoconnectivity** | | | **hyperconnectivity** | | |
| **region** | **CS** | **rank** | **region** | **CS** | **rank** | **region** | **CS** | **rank** | **region** | **CS** | **rank** |
| Precuneus_L | 42 | 1 | Frontal_Inf_Tri_L | 15 | 1 | Precentral_R | 34 | 1 | Temporal_Pole_Mid_R | 20 | 1 |
| Precuneus_R | 39 | 2 | Frontal_Inf_Oper_L | 10 | 2 | Postcentral_L | 32 | 2 | Frontal_Inf_Tri_R | 17 | 2 |
| Paracentral_Lobule_R | 38 | 3 | Frontal_Inf_Tri_R | 9 | 3 | Precentral_L | 31 | 3 | Frontal_Inf_Oper_R | 16 | 3 |
| Angular_L | 35 | 4 | Temporal_Pole_Sup_L | 7 | 4 | Postcentral_R | 30 | 4 | Frontal_Inf_Orb_L | 14 | 4 |
| Cingulum_Mid_R | 35 |  | Insula_L | 7 |  | Temporal_Pole_Sup_L | 25 | 5 | Frontal_Mid_R | 14 |  |
| Calcarine_L | 33 | 5 | Temporal_Pole_Mid_R | 6 | 5 | Temporal_Pole_Sup_R | 24 | 6 | Frontal_Sup_R | 13 | 5 |
| Parietal_Inf_R | 32 | 6 | Hippocampus_L | 6 |  | Frontal_Inf_Oper_L | 23 | 7 | Temporal_Pole_Sup_R | 12 | 6 |
| Lingual_R | 32 |  | Temporal_Inf_L | 6 |  | Cingulum_Mid_L | 22 | 8 | Frontal_Inf_Tri_L | 12 |  |
| Occipital_Mid_R | 32 |  | Rolandic_Oper_R | 6 |  | Frontal_Inf_Orb_L | 22 |  | Amygdala_L | 12 |  |
| Cingulum_Mid_L | 29 | 7 | ParaHippocampal_L | 6 |  | Rolandic_Oper_L | 20 | 9 | Amygdala_R | 12 |  |
| Angular_R | 29 |  | Temporal_Sup_R | 6 |  | Temporal_Sup_R | 19 | 10 | Temporal_Sup_R | 11 | 7 |
| Occipital_Mid_L | 28 | 8 | Heschl_R | 6 |  | Rectus_R | 19 |  | Temporal_Mid_R | 11 |  |
| Lingual_L | 28 |  | Cingulum_Post_L | 5 | 6 | Rolandic_Oper_R | 18 | 11 | Rolandic_Oper_R | 10 | 8 |
| Rolandic_Oper_L | 27 | 9 | Amygdala_R | 5 |  | Rectus_L | 18 |  | Temporal_Pole_Mid_L | 10 |  |
| Cingulum_Post_R | 27 |  | Temporal_Inf_R | 5 |  | Temporal_Sup_L | 18 |  | Temporal_Pole_Sup_L | 9 | 9 |
| Parietal_Sup_R | 27 |  | Precentral_R | 5 |  | SupraMarginal_L | 18 |  | Heschl_R | 9 |  |
| Temporal_Pole_Sup_L | 26 | 10 | Frontal_Sup_R | 5 |  | Frontal_Sup_Medial_R | 18 |  | Frontal_Sup_L | 9 |  |
| Temporal_Pole_Mid_L | 26 |  | Hippocampus_R | 5 |  | Heschl_R | 17 | 12 | Occipital_Inf_L | 9 |  |
| Temporal_Pole_Mid_R | 25 | 11 | Frontal_Inf_Oper_R | 5 |  | Frontal_Inf_Tri_L | 16 | 13 | Frontal_Inf_Orb_R | 9 |  |
| Hippocampus_L | 24 | 12 | Frontal_Mid_L | 5 |  | Insula_L | 15 | 14 | Cuneus_R | 9 |  |
| Occipital_Sup_L | 24 |  | ParaHippocampal_R | 5 |  | Frontal_Mid_R | 14 | 15 | Angular_R | 9 |  |
| Heschl_L | 22 | 13 | Cingulum_Mid_R | 4 | 7 | Parietal_Inf_L | 14 |  | Lingual_R | 9 |  |
| Cingulum_Post_L | 22 |  | Cingulum_Mid_L | 4 |  | Frontal_Sup_L | 13 | 16 | Frontal_Mid_L | 9 |  |
| Insula_L | 21 | 14 | Cingulum_Post_R | 4 |  | Heschl_L | 13 |  | Frontal_Sup_Medial_L | 9 |  |
| Cuneus_L | 21 |  | Paracentral_Lobule_L | 4 |  | Insula_R | 12 | 17 | Frontal_Med_Orb_L | 9 |  |
| Occipital_Inf_R | 21 |  | Temporal_Mid_R | 4 |  | Occipital_Mid_L | 12 |  | Insula_L | 8 | 10  10  10  10  10  10  10 |
| Parietal_Sup_L | 21 |  | Cuneus_R | 4 |  | ParaHippocampal_L | 11 | 18 | Insula_R | 8 |  |
| SupraMarginal_L | 20 | 15 | Supp_Motor_Area_R | 4 |  | Occipital_Inf_L | 11 |  | Cingulum_Post_R | 8 |  |
| Occipital_Inf_L | 20 |  | Frontal_Mid_R | 4 |  | Cingulum_Post_L | 10 | 19 | Parietal_Inf_R | 8 |  |
| Paracentral_Lobule_L | 20 |  | Calcarine_L | 3 | 8 | Cingulum_Post_R | 10 |  | Precuneus_R | 8 |  |
| Temporal_Inf_L | 19 | 16 | Occipital_Mid_R | 3 |  | Cingulum_Ant_R | 10 |  | Frontal_Sup_Orb_L | 8 |  |
| Fusiform_L | 19 |  | Temporal_Pole_Mid_L | 3 |  | Frontal_Sup_R | 9 | 20 | ParaHippocampal_R | 8 |  |
| Amygdala_R | 19 |  | Postcentral_L | 3 |  | Frontal_Inf_Oper_R | 9 |  | Postcentral_R | 7 | 11  11  11  11  11  11  11  11 |
| Postcentral_L | 17 | 17 | Temporal_Mid_L | 3 |  | Supp_Motor_Area_R | 9 |  | Hippocampus_L | 7 |  |
| Temporal_Mid_R | 17 |  | Fusiform_R | 3 |  | Cuneus_L | 9 |  | Supp_Motor_Area_L | 7 |  |
| Temporal_Inf_R | 17 |  | Olfactory_L | 3 |  | Angular_L | 9 |  | Temporal_Inf_R | 7 |  |
| Rolandic_Oper_R | 16 | 18 | Rectus_L | 3 |  | Frontal_Inf_Tri_R | 8 | 21 | Olfactory_L | 7 |  |
| Postcentral_R | 15 | 19 | Frontal_Inf_Orb_L | 3 |  | Hippocampus_L | 8 |  | Parietal_Sup_R | 7 |  |
| Temporal_Sup_L | 15 |  | Temporal_Pole_Sup_R | 3 |  | Temporal_Inf_L | 8 |  | Cingulum_Ant_L | 7 |  |
| Parietal_Inf_L | 15 |  | Insula_R | 3 |  | Frontal_Inf_Orb_R | 8 |  | Frontal_Sup_Orb_R | 7 |  |
| ParaHippocampal_L | 15 |  | Frontal_Sup_L | 3 |  | Precuneus_L | 8 |  | Postcentral_L | 6 | 12  12  12  12  12  12  12  12  12  12  12 |
| Rectus_R | 14 | 20 | Cingulum_Ant_L | 3 |  | Temporal_Mid_R | 7 | 22 | Precentral_L | 6 |  |
| Temporal_Mid_L | 14 |  | Lingual_L | 2 | 9 | Amygdala_L | 7 |  | Occipital_Mid_L | 6 |  |
| Fusiform_R | 14 |  | Cuneus_L | 2 |  | Frontal_Mid_Orb_R | 7 |  | Frontal_Mid_Orb_R | 6 |  |
| SupraMarginal_R | 13 | 21 | Fusiform_L | 2 |  | Hippocampus_R | 6 | 23 | SupraMarginal_R | 6 |  |
| Olfactory_L | 13 |  | Rectus_R | 2 |  | Calcarine_L | 6 |  | Frontal_Mid_Orb_L | 6 |  |
| Occipital_Sup_R | 13 |  | Cingulum_Ant_R | 2 |  | Fusiform_L | 6 |  | Occipital_Sup_L | 6 |  |
| Rectus_L | 12 | 22 | Precentral_L | 2 |  | Supp_Motor_Area_L | 6 |  | Occipital_Sup_R | 6 |  |
| Calcarine_R | 12 |  | Amygdala_L | 2 |  | Parietal_Inf_R | 6 |  | Calcarine_R | 6 |  |
| Precentral_R | 11 | 23 | Supp_Motor_Area_L | 2 |  | Occipital_Inf_R | 6 |  | Occipital_Mid_R | 6 |  |
| Frontal_Inf_Orb_L | 11 | 24 | Frontal_Sup_Orb_L | 2 |  | SupraMarginal_R | 6 |  | Olfactory_R | 6 |  |
| Temporal_Pole_Sup_R | 10 | 25 | Frontal_Sup_Orb_R | 2 |  | Amygdala_R | 5 | 24 | Frontal_Inf_Oper_L | 5 | 13 |
| Temporal_Sup_R | 9 | 26 | Frontal_Inf_Orb_R | 2 |  | Temporal_Inf_R | 5 |  | Rectus_R | 5 |  |
| Cuneus_R | 9 |  | Frontal_Mid_Orb_L | 2 |  | Cuneus_R | 5 |  | Rectus_L | 5 |  |
| Frontal_Inf_Oper_L | 8 | 27 | Frontal_Sup_Medial_L | 2 |  | Lingual_L | 5 |  | Temporal_Sup_L | 5 |  |
| Heschl_R | 8 |  | Angular_L | 1 | 10 | Frontal_Mid_Orb_L | 5 |  | Frontal_Sup_Medial_R | 5 |  |
| Cingulum_Ant_R | 8 |  | Rolandic_Oper_L | 1 |  | Occipital_Sup_L | 5 |  | ParaHippocampal_L | 5 |  |
| Precentral_L | 7 | 28 | Occipital_Sup_L | 1 |  | Angular_R | 5 |  | Supp_Motor_Area_R | 5 |  |
| Insula_R | 7 |  | Heschl_L | 1 |  | Temporal_Pole_Mid_R | 4 | 25 | Fusiform_L | 5 |  |
| Amygdala_L | 7 |  | Parietal_Sup_L | 1 |  | Cingulum_Mid_R | 4 |  | Cingulum_Ant_R | 4 | 14 |
| Supp_Motor_Area_L | 7 |  | Occipital_Inf_L | 1 |  | Olfactory_L | 4 |  | Cuneus_L | 4 |  |
| Frontal_Sup_Orb_L | 7 |  | Postcentral_R | 1 |  | Occipital_Sup_R | 4 |  | Precuneus_L | 4 |  |
| Frontal_Sup_Orb_R | 7 |  | Temporal_Sup_L | 1 |  | Lingual_R | 4 |  | Calcarine_L | 4 |  |
| Frontal_Sup_Medial_R | 6 | 29 | Parietal_Inf_L | 1 |  | Parietal_Sup_R | 4 |  | Cingulum_Mid_R | 4 |  |
| Frontal_Inf_Orb_R | 6 |  | Occipital_Sup_R | 1 |  | Paracentral_Lobule_L | 3 | 26 | Fusiform_R | 4 |  |
| Frontal_Med_Orb_L | 6 |  | Calcarine_R | 1 |  | Temporal_Mid_L | 3 |  | Precentral_R | 3 | 15 |
| Frontal_Sup_R | 5 | 30 | Precuneus_L | 0 | 11 | Cingulum_Ant_L | 3 |  | Rolandic_Oper_L | 3 |  |
| Hippocampus_R | 5 |  | Precuneus_R | 0 |  | Calcarine_R | 3 |  | Heschl_L | 3 |  |
| Frontal_Inf_Oper_R | 4 | 31 | Paracentral_Lobule_R | 0 |  | Paracentral_Lobule_R | 3 |  | Cingulum_Post_L | 3 |  |
| Frontal_Mid_Orb_R | 4 |  | Parietal_Inf_R | 0 |  | Frontal_Med_Orb_R | 3 |  | Angular_L | 3 |  |
| Frontal_Mid_Orb_L | 4 |  | Lingual_R | 0 |  | Frontal_Mid_L | 2 | 27 | Lingual_L | 3 |  |
| Frontal_Sup_L | 3 | 32 | Angular_R | 0 |  | Occipital_Mid_R | 2 |  | Paracentral_Lobule_L | 3 |  |
| Frontal_Inf_Tri_R | 3 |  | Occipital_Mid_L | 0 |  | Precuneus_R | 2 |  | Temporal_Mid_L | 3 |  |
| Cingulum_Ant_L | 3 |  | Parietal_Sup_R | 0 |  | Temporal_Pole_Mid_L | 1 | 28 | Cingulum_Mid_L | 2 | 16 |
| Supp_Motor_Area_R | 1 | 33 | Occipital_Inf_R | 0 |  | Fusiform_R | 1 |  | SupraMarginal_L | 2 |  |
| Frontal_Med_Orb_R | 1 |  | SupraMarginal_L | 0 |  | Frontal_Sup_Orb_L | 1 |  | Parietal_Inf_L | 2 |  |
| Frontal_Sup_Medial_L | 1 |  | SupraMarginal_R | 0 |  | Frontal_Sup_Orb_R | 1 |  | Temporal_Inf_L | 2 |  |
| Olfactory_R | 1 |  | Frontal_Sup_Medial_R | 0 |  | Frontal_Sup_Medial_L | 1 |  | Hippocampus_R | 2 |  |
| Frontal_Inf_Tri_L | 0 | 34 | Frontal_Med_Orb_L | 0 |  | Parietal_Sup_L | 1 |  | Occipital_Inf_R | 2 |  |
| Frontal_Mid_R | 0 |  | Frontal_Mid_Orb_R | 0 |  | Frontal_Med_Orb_L | 1 |  | Frontal_Med_Orb_R | 2 |  |
| Frontal_Mid_L | 0 |  | Frontal_Med_Orb_R | 0 |  | ParaHippocampal_R | 0 | 29 | Parietal_Sup_L | 2 |  |
| ParaHippocampal_R | 0 |  | Olfactory_R | 0 |  | Olfactory_R | 0 |  | Paracentral_Lobule_R | 1 | 17 |

CS: consistency score, rank: ranking of the regions according to the consistency score

##### Figure S1


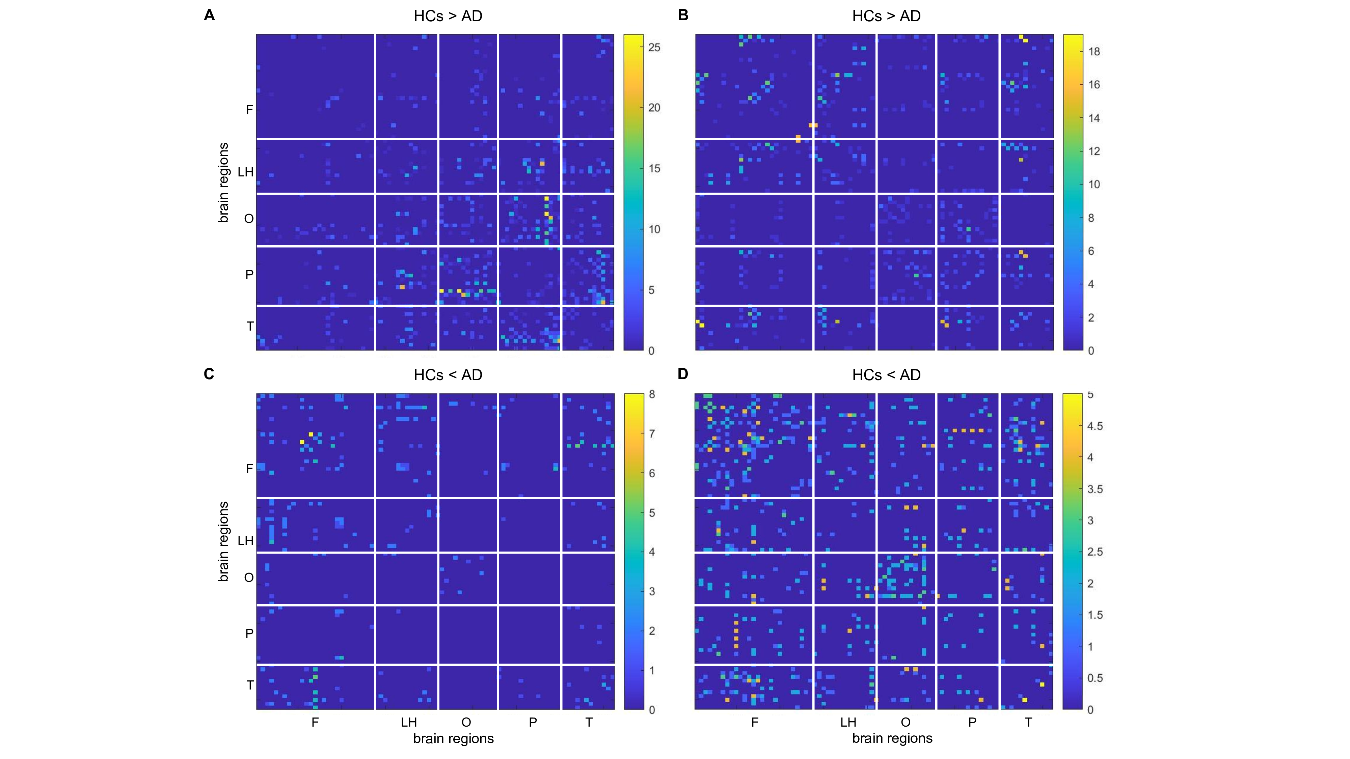


**Figure S1:** Representativity of pairwise functional interactions with statistically significant differences between healthy controls (HCs) and each dementia subtyps (Alzheimer’s disease, AD; and behavioral variant frontotemporal dementia, bvFTD). The colors represent the number of types of connectivity (types of functional interactions) at which statistical differences were obtained. Each type of connectivity corresponds to either a time-domain interaction or a frequency-domain interaction computed in a particular frequency band of the EEG. Connections with increased functional connectivity in dementia relative to HCs are presented for **A)** patients with AD, and **B)** patients with bvFTD. Connections with increased functional connectivity in dementia relative to HCs are presented for **C)** AD, and **D)** bvFTD. Brain regions are sorted following the order of the automatic anatomical labeling (AAL) atlas, and correspond to the frontal lobe (F), the limbic lobe plus hippocampal-related regions (LH), the occipital lobe (O), the parietal lobe (P), and the temporal lobe (T).

##### Figure S2


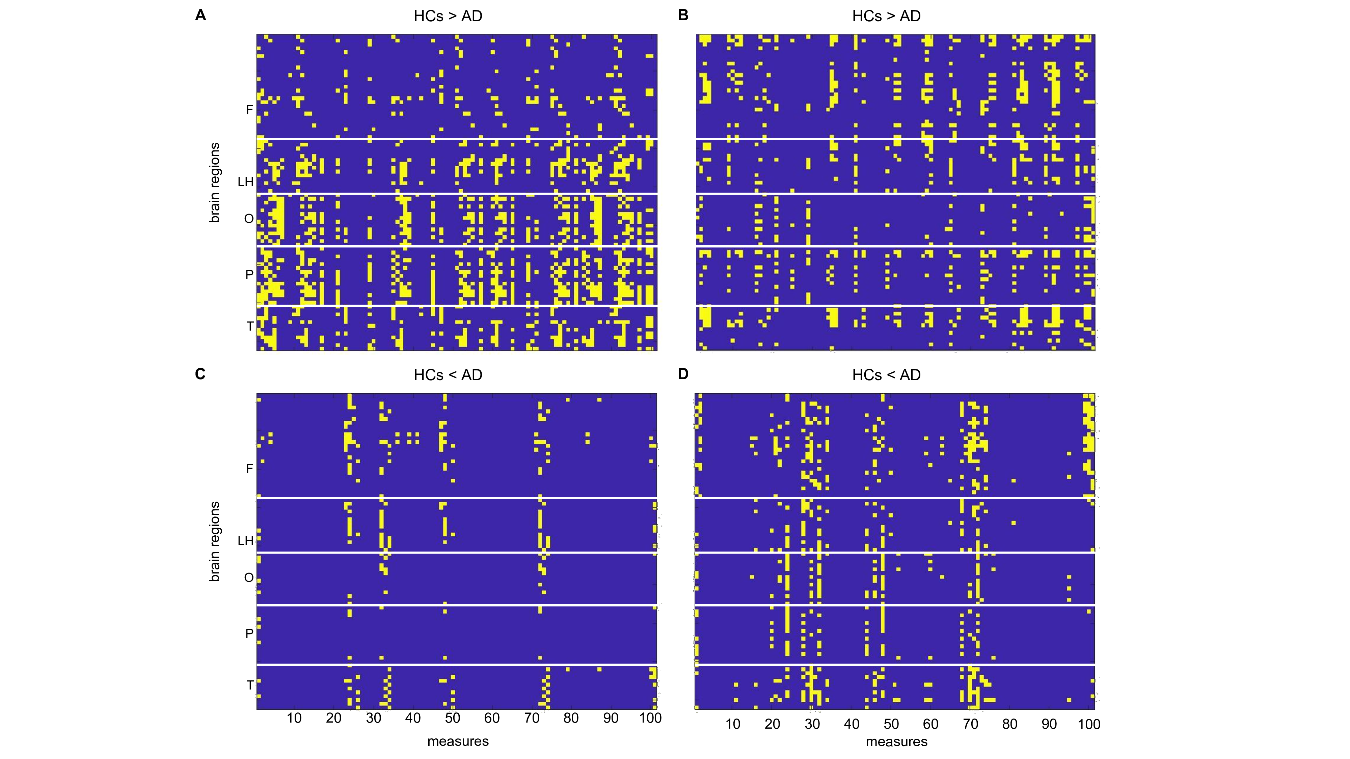


**Figure S2:** Atypical functional connectivity in dementia can be assessed by different connectivity measures. Regions with increased functional connectivity relative to healthy controls (HCs) are presented in for **A)** patients with Alzheimer’s disease (AD), and **B)** patients with behavioral variant frontotemporal dementia (bvFTD). Likewise, regions with increased functional connectivity relative to HCs are presented for **C)** AD, and **D)** bvFTD. Numbers in the abscissa axis (x) represent different types of functional interactions, which in turn correspond to either a time-domain interaction or a frequency-domain interaction computed in a particular frequency band of the EEG. The order in which they are presented in this figure is provided in table S4. Functional connectivity is presented in binary matrices. Therefore, this representation only considers if brain regions displayed altered connectivity, but does not inform about the number of altered pairwise connections to which they belong. Brain regions are sorted following the order of automatic anatomical labeling (AAL) atlas. Brain regions correspond to the frontal lobe (F), the limbic lobe plus hippocampal related regions (LH), the occipital lobe (O), the parietal lobe (P), and the temporal lobe (T).

#####
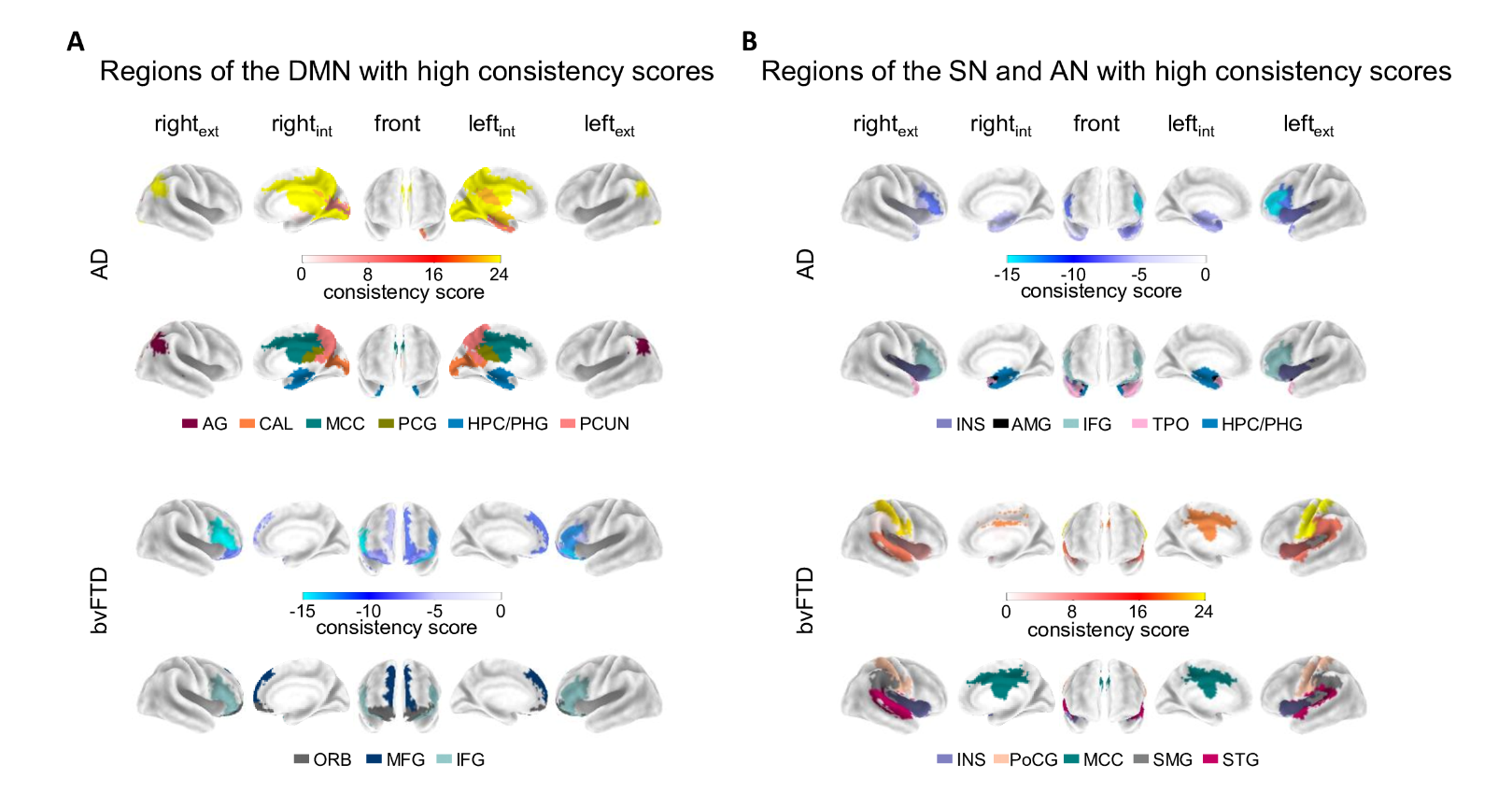
Figure S3

**Figure S3:** Atypical functional connectivity of relevant nodes of resting-state functional networks in dementia. The divergent functional connectivity patterns of the default mode network (DMN) and the salience network (SN) in Alzheimer’s disease (AD) and behavioral variant frontotemporal dementia (bvFTD) is illustrated. The SN is presented in association with the auditory network (AN). **A)** Nodes of the DMN with high consistency score. **B)** Nodes of the SN with high consistency score. In each case, regions belong to the set of connections that contribute most to the classification models (connection with high SHAP values). For illustrative purposes, panels presenting the consistency scores were accompanied by panels showing the topographic information of the brain regions. Positive and negative consistency scores indicate hypo- and hyperconnectivity, respectively. Noteworthy, consistency scores are the same as presented in Figure 1C and Table S2. ORB: orbital cortex, MFG: middle frontal gyrus, IFG: inferior frontal gyrus, PCG: precentral gyrus, MCC: middle cingulate cortex, HP: hippocampus, PHG: Parahippocampal gyrus, INS: insula, AMG: amygdala, TPO: temporal pole, STG: superior temporal gyrus, AG: Angular Gyrus, PCUN: precuneus, PoCG: postcentral gyrus, SMG: supramarginal gyrus, CAL: calcarine sulcus and surrounding cortices.
